# Supplementary material for: Geography-independent mucosal microbiota alterations in primary sclerosing cholangitis persist after liver transplantation
Source: JHEP Rep. 2025 Dec 22;8(4):101716. doi: 10.1016/j.jhepr.2025.101716 (PMC12972987; doi:10.1016/j.jhepr.2025.101716)
Supplement: Multimedia component 1 [file mmc1.pdf]

Geography-independent mucosal microbiota alterations in primary sclerosing cholangitis persist after liver transplantation

Lukas Bajer, Petra Polakovicova, Marie Heczkova, Kristian Holm, Mikal J. Hole, Mojmir Hlavaty, Alena Bohdanecka, Pavel Drastich, Filip Tichanek, Malin H. Meyer-Myklestad, Asle W. Medhus, Dag Henrik Reikvam, Kristin K. Jørgensen, Jan Brezina, Peter Macinga, Pavel Wohl, Ondrej Fabian, Johannes R. Hov, Monika Cahova

Table of contents

Supplementary methods…………………………………………………………..…...2

Supplementary figures…………………………………………………………..……7

Supplementary tables…………………………………………………………..…….9

Supplementary references……………………………………………………………10

Supplementary methods

Study Population

This study is based on two cohorts from Czech Republic and Norway, which consist of 115 non-transplanted PSC (pre_LTx), 159 transplanted PSC (post_LTx) patients, and 96 healthy controls (HCs) Czech subjects underwent colonoscopy between 2021 and 2023 at IKEM, while Norwegian subjects underwent colonoscopy between 2005 and 2008 at Rikshospitalet, Oslo University Hospital. Diagnoses of PSC and IBD were made according to clinical guidelines[1, 2](Karlsen, Lennard). The diagnosis of rPSC was made when cholangiography or histology findings consistent with PSC were present in the absence of defined causes of secondary sclerosing cholangitis[3, 4](Lindstrom, Graziadei). Multiple samples were collected from different gut sites: the terminal ileum in both Czech and Norwegian cohorts, the cecum and rectum in the Czech cohort, and the colon ascendens, colon descendens, and colon sigmoideum in the Norwegian cohorts. For the purpose of comparison and merging of these cohorts, the colon segments were unified under one term - colon. Revised Mayo PSC risk score, Amsterdam–Oxford model (AOM) score for PSC, the aspartate aminotransferase (AST) to platelet ratio index (APRI), and the Fibrosis-4 score (FIB-4) were calculated according to earlier works[5-8] (deVries, Sterling, Wai, Kim). Details are given in **Table 1** and **Supplementary Table 1**.

Sample collection, storage, library preparation, and sequencing

Mucosal biopsies were sampled using standard forceps. PSC samples from the Norwegian cohort were snap-frozen in dry tubes and stored without preservatives, while biopsies from HCs were preserved in RNAlater (ThermoFisherScientific, Waltham, MA); Czech samples were preserved by DNA/RNA Shield (Zymoresearch, Irvine, California, USA). All samples were stored at −80°C until analysis. Fecal DNA from samples in the Czech cohort was isolated by QIAmp PowerFecal DNA Kit (Qiagen), while for Norwegian samples, All Prep DNA/RNA mini kit (Qiagen, Hilden, Germany) was used. Isolated DNA was used as a template in PCR reactions targeting the V3–V4 hypervariable regions of the 16S rRNA gene, using primers 319F/806R for Norwegian samples and 341F/806R for Czech samples, respectively, along with either Q5 High-Fidelity 2× Master Mix (BioLabs, New England) or Phusion High-Fidelity PCR Master Mix with HF buffer. A dual-indexing approach was used. PCR products from Czech samples were cleaned using SPRIselect beads (Beckman Coulter Genomics) and pooled equimolarly according to measured concentrations. PCR products from Norwegian samples were cleaned and normalized using the SequalPrep Normalization Plate Kit (Thermo Fisher Scientific). Quality control and quantification of pooled libraries were performed using an Agilent Bioanalyzer (Agilent Technologies, Santa Clara, CA). Sequencing of Czech DNA libraries was conducted at the Biocev Core Facility (OMICS-Genomics Biocev, Vestec, Czech Republic) on the Illumina MiSeq platform (2×250 bp). Sequencing of Norwegian DNA libraries was performed at the Norwegian Sequencing Centre (Oslo, Norway) on the Illumina MiSeq platform (2×300 bp).

Bioinformatics processing

The Illumina paired-end reads were first quality-checked using FastQC v0.11.9 (https://www.bioinformatics.babraham.ac.uk/projects/fastqc/) and MultiQC v1.12[9]. Nextera Transposase Adapters and PhiX sequences were discarded using BBDuk v39.06 (https://doi.org/10.14806/ej.17.1.200), primers were trimmed by cutadapt v4.9. The reads were then merged using BBMerge v39.06 and oriented with VSEARCH v2.28.1[10]. Amplicon sequence variants (ASVs) were obtained using deblur in QIIME2 version 2024.2[11], after initial trimming to 400bp. The amplicon-region-specific sklearn-based Naive Bayes classifier was trained based on the SILVA reference database[12], release 138.1, with representative sequences at 99% identity via RESCRIPt QIIME 2 plugin. These steps were conducted on a per-run basis, followed by merging the run results using QIIME's feature-table merge, merge-seqs, and merge-taxa functions. Finally, mitochondrial and

chloroplast sequences were filtered out based on the obtained taxonomic assignment. Scripts used to process the raw sequencing data are available at https://github.com/xpolak37/PSC_study.

Statistical analysis

All statistical analyses were conducted using R v4.3.1. The Czech and Norwegian cohorts were merged at the ASV level using custom functions and then divided into two segments: terminal ileum and colon. As the first step, post_LTx, pre_LTx, and HCs samples were analyzed. In the second step, the post_LTx group was further divided into rPSC and non-rPSC groups, with healthy controls also being analyzed. An IBD vs. no-IBD comparison was performed within PSC patients (pre-LTx and rPSC individuals combined). In each step, alpha diversity, beta diversity, differential abundance analysis (DAA) were conducted, and a binary classifier was trained to assess the discriminating power between groups. For the purpose of further calculations, ASVs were aggregated to genus level except alpha diversity, which was calculated at ASV level only. All scripts and analysis reports are available at https://github.com/xpolak37/PSC_study.

Filtering

A rarefaction curve was constructed to determine and visualize suitable sequencing depth for rarefaction using phyloseq package v1.46.0[13]. This was set at 10,000 reads; therefore, all samples below this sequencing depth were removed. The nearZeroVar() function from caret package v6.0-94[14] with default parameters was used to filter low-prevalent and low-abundant taxa. This filtering step was applied to beta diversity, DAA, and classification by the machine learning model, but not for alpha diversity calculations.

Alpha Diversity

Alpha diversity (ASV Richness and Shannon indexes) was calculated on rarefied data at the ASV level. Rarefaction and alpha diversity calculation were performed using the MicrobiotaProcess package v1.14.1[15]. To test differences between groups in the terminal ileum, a linear fixed-effects model was applied (stats package v4.3.1) that accounted for the effect of Country and its interaction with the Group. In the colon dataset, a linear mixed-effect model (robustlmm package v3.3.1)[16] was used, with the Patient variable included as a random effect to account for multiple samples from the same patient, due to merging several segments into a single colon segment. The Benjamini-Hochberg (BH) correction was applied to control the false discovery rate (FDR) for multiple comparisons.

Beta Diversity

The Aitchison distance was calculated using the 'robust_aitchison' method with the vegdist() function in the vegan v2.6.4 package. Permutational MANOVA (PERMANOVA) was performed using the adonis2() (vegan v2.6.4) to assess the effects of Group and Cohort. Initially, the main effects of Group and Cohort were tested using the *by = 'margin'* setting. To evaluate the interaction effect, the model was run once more with *by = 'terms'* setting. When the interaction effect was significant, post-hoc analysis was conducted by separately testing the effect of Group within each Cohort and the effect of Cohort within each Group. In the terminal ileum dataset, PERMANOVA was performed with 999 free permutations. In the colon dataset, 999 custom permutations were used, ensuring that each patient remained assigned to the same group across samples. The BH correction was applied to control the FDR for multiple comparisons. Beta diversity of individual groups was visualized using principal coordinate analysis (PCoA) based on robust Aitchison distance.

Differential Abundance Analysis

Two tools were used for DAA to minimize false positives: linDA from the MicrobiomeStat package v1.2[17] and the Maaslin2 package v1.16.0[18]. The intersection of these two tools was used to identify differentially abundant taxa between groups. From this list, taxa with a significant interaction effect

were excluded based on post-hoc analysis of the Czech and Norwegian cohorts individually. Only taxa with significant log fold change that showed the same direction of change in both cohorts were retained. Taxa associated with the PSC effect were identified by intersecting the differentially abundant taxa from the comparisons between pre_LTx and healthy groups and between post_LTx and healthy groups. Statistics for individual taxa are reported as quartiles of relative abundances for each group and cohort in **Supplementary Tables 2 and 3**. **Supplementary Tables 7 and 8** list the results of DAA, specifically the log fold change, p-values, and FDR values calculated by linDA for each tested comparison.

Classification

Binary classification was performed using Elastic Net (ENET) with glmnet v4.1.8[19] and three supplementary models: Random Forest (RF) with ranger v0.17.0[20], Gradient Boosting (GBoost) with gbm v2.2.2, and K-nearest Neighbors (kNN) with kknn v1.3.1[21]. Hyperparameter tuning was conducted for each model using five-fold cross-validation to estimate the optimal parameters. For the ENET model, the cv.glmnet() function was used to determine the optimal alpha and lambda values (lambda.1se was used). In the other models, the optimal parameters were found using grid search from the caret package. For the GBoost model, tuning was performed for the number of trees (ntrees), interaction depth, shrinkage, and the minimum number of observations in a node (n.minobsinnode). For the kNN classifier, the optimal number of neighbors (k) was tuned. Lastly, the optimal number of variables randomly sampled as candidates at each split (mtry), the minimum node size (min.node.size) was tuned in the RF model. The splitting criterion was set to Gini impurity (splitrule). The tuned parameters, obtained by fitting on the entire dataset, are listed bellow. When validating the models though bootstrapping, the parameters were re-tuned in each iteration.

		ENET		kNN	RF		Gboost			
		alpha	lambda	k	mtry	Min node size	n.trees	interaction depth	shrinkage	n.minobsin node
pre_LTx vs HC	terminal ileum	0.20	0.040	12	47	5	500	1	0.1	20
pre_LTx vs post_LTx		0.00	0.667	17	57	2	100	1	0.1	10
post_LTx vs HC		0.00	0.071	12	133	2	200	3	0.1	20
rPSC vs non-rPSC		0.4	0.312	30	137	2	200	3	0.1	20
rPSC vs non-rPSC (subset)		0.2	0.502	30	19	5	100	1	0.1	20
pre_LTx vs HC	colon	0.00	0.043	26	23	2	500	5	0.1	20
pre_LTx vs post_LTx		0.00	0.052	15	71	2	500	5	0.1	10
post_LTx vs HC		0.60	0.002	30	65	2	500	3	0.1	10
rPSC vs non-rPSC		0	0.036	12	61	2	500	3	0.1	10
rPSC vs non-rPSC (subset)		0.8	0.033	11	13	2	500	3	0.1	20

These models were trained and validated through bootstrapping (N=500) on CLR-transformed data. Model performance metrics were calculated based on an out-of-bag principle. Bootstrap samples were obtained using sample() function with replacement, with set seed to ensure reproducible results. In the colon dataset, the bootstrap dataset was pseudo-randomly generated to ensure that multiple samples from a single patient were kept in either the training or validation dataset. In all cases, the model was trained using all taxa that remained after preprocessing. For the second objective, distinguish between rPSC and non-rPSC samples, an additional model was trained using only the taxa associated with the rPSC effect identified through DAA. This approach was intended to verify the accurate identification of these taxa and to enhance the model's classification performance.

Classifier performance was evaluated using accuracy metric and the Area Under the Curve (AUC) metric from the Receiver Operating Characteristic (ROC) curve calculated using the pROC package v1.18.5[22]. Optimism-corrected AUC was estimated as the mean AUC from the validation performance across the bootstrapped samples. The lower and upper confidence intervals were calculated using the 2.5th and 97.5th percentiles of the validation AUC distribution. To prevent potential training errors and overfitting, a validity check was performed for all models by randomly shuffling the sample labels. The performance of these models should not reach significantly high values. As expected, the AUC did not exceed 0.57.

Microbial Dysbiosis Index

The Microbial Dysbiosis index (MDI) was defined as clr(total abundance of taxa increased in PSC) – clr(total abundance of taxa decreased in PSC), separately for ileum and colon samples at genus level. Differences between groups were tested using a linear fixed-effects model in the terminal ileum and a linear mixed-effects model in the colon. In the mixed-effects model, the 'Patient' variable was included as a random effect to account for multiple samples from the same patients, same approach like in alpha diversity analysis.

Correlation Analysis

Clinical parameters associated with the severity of gut and liver conditions, as well as other relevant factors, were selected for analysis. The Spearman's correlation coefficient was calculated between the MDI and clinical parameters to assess the relationship between the gut and liver disease severity. For the colon samples, the correlation was computed 100 times, with each calculation performed on a randomly selected sample from each patient. The final reported correlation represents the average across all iterations. A correlation was considered significant if at least 90 out of the 100 iterations yielded a p value < 0.05.

Supplementary figures

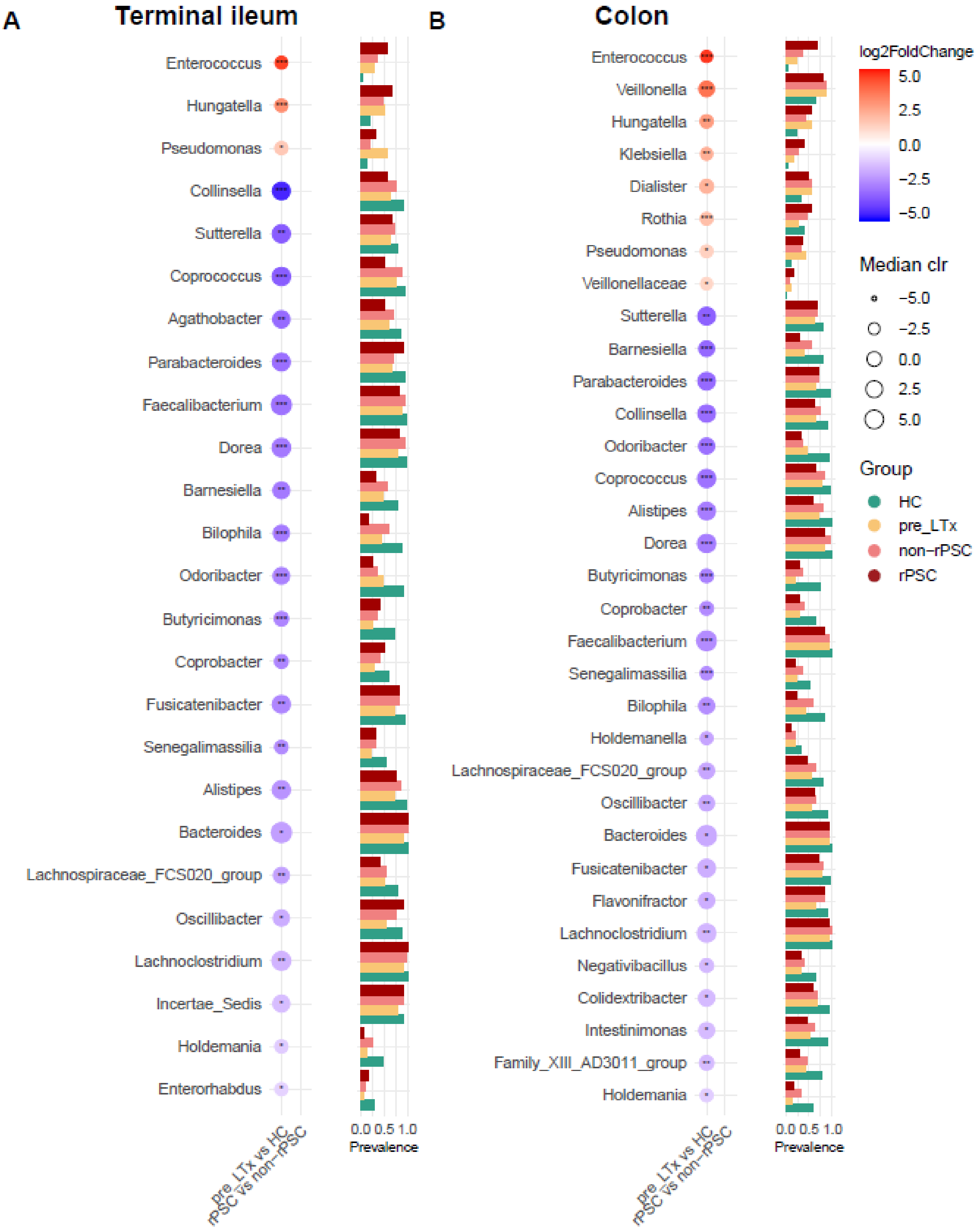

Fig. S1 *Comparison of the relative abundance of bacterial taxa: pre_LTx, post_LTx_rPSC, post_LT_non-rPSC, and HC groups.* (A) Terminal ileum; (B) Colon. The differential abundance analysis was performed independently by two tools (linDA and MaAslin 2), and only the intersection of sets selected by each tool was shown to minimize false positives. The prevalence is calculated as a ratio of samples in which the particular taxon is present to the total number of samples in the group. * q<0.05; * q<0.05; ***q<0.001.

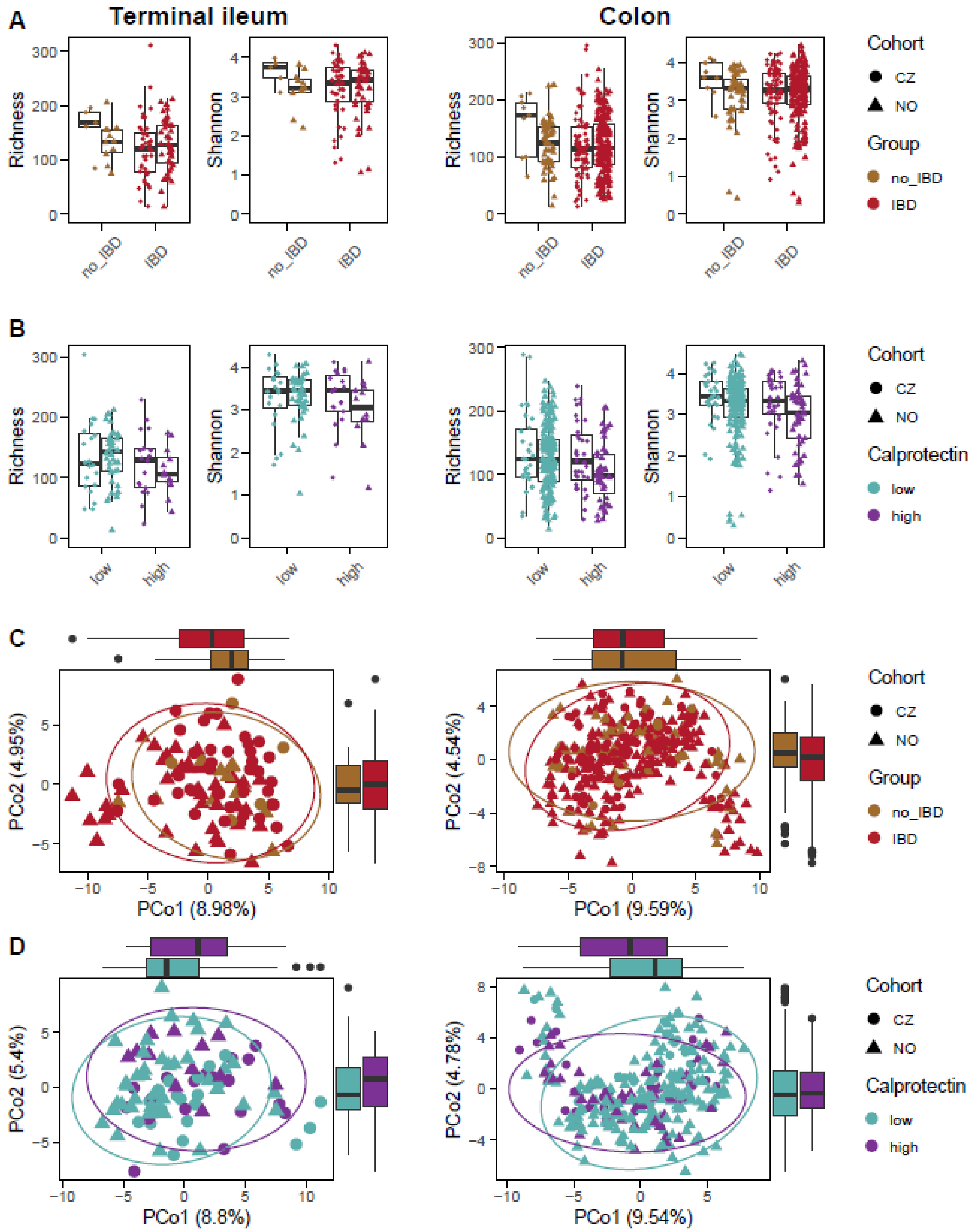

Fig. S2 *Relationship between intestinal inflammation, PSC status and microbiota.* Alpha diversity in the terminal ileum and colon assessed by the Richness and the Shannon indexes based on amplicon sequence variants (ASVs) in (A) IBD and non-IBD groups; (B) low- and high inflammatory groups. Principal coordinate plots showing microbiota composition in (C) IBD and non-IBD groups and (D) low- and high-inflammatory groups. The low and high-inflammatory groups were defined based on fecal calprotectin concentration at a threshold of 250 μg/g.

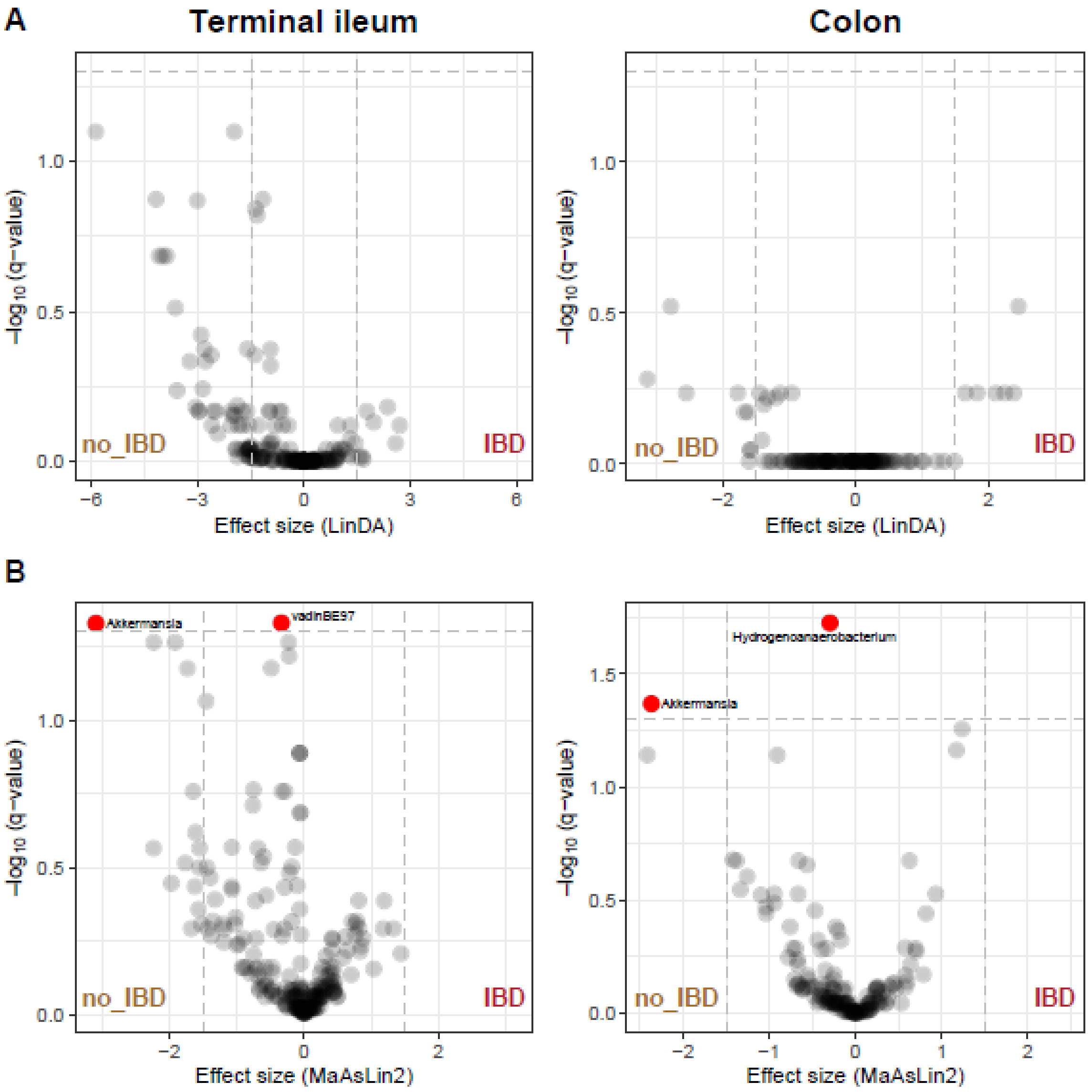

Fig. S3 *Effect of intestinal inflammation on microbiota composition in PSC in terminal ileum and colon.* (A) Volcano plots generated using linDA; (B) Volcano plot generated using MaAsLin2. For this analysis, pre-LTx and post_LTx rPSC patients were pooled and divided according to the history of IBD diagnosis into the PSC_IBD and PSC_noIBD groups. Negative effect size is associated with higher abundance in PSC patients without IBD. The significance threshold was set at q<0.05.

Supplementary tables

Available at https://doi.org/10.6084/m9.figshare.29430899

Table S1 Cohort overview
Table S2 Composition of mucosal microbiota in ileum (genus level).
Table S3 Composition of mucosal microbiota in colon (genus level). Table S4 *Effect of the group and cohort on alpha diversity* Table S5 *PERMANOVA results.*
Table S6 *The predictive power of microbiome features to identify the group* Table S7 *PSC-associated mucosal microbiota: terminal ileum* Table S8 *PSC-associated mucosal microbiota: colon*
Table S9 *Effect of the group on alpha and beta diversity: ALD vs PSC post_LTx patients*
Table S10 *Effect of the group and cohort on alpha diversity*
Table S11 *PERMANOVA results*
Table S12 *The predictive power of microbiome features to identify the group*

Supplementary references

1. Karlsen TH, Folseraas T, Thorburn D, et al. Primary sclerosing cholangitis–a comprehensive review. Journal of hepatology 2017;67:1298-1323.
2. Lennard-Jones J. Classification of inflammatory bowel disease. Scandinavian Journal of Gastroenterology 1989;24:2-6.
3. Lindström L, Jørgensen KK, Boberg KM, et al. Risk factors and prognosis for recurrent primary sclerosing cholangitis after liver transplantation: a Nordic Multicentre Study. Scandinavian journal of gastroenterology 2018;53:297-304.
4. Graziadei IW, Wiesner RH, Batts KP, et al. Recurrence of primary sclerosing cholangitis following liver transplantation. Hepatology 1999;29:1050-1056.
5. de Vries EM, Wang J, Williamson KD, et al. A novel prognostic model for transplant-free survival in primary sclerosing cholangitis. Gut 2018;67:1864-1869.
6. Sterling R, Lissen E, Clumeck N, et al. S Sulkowski M, Torriani FJ, Dieterich DT, Thomas DL, Messinger D, Nelson M; APRICOT Clinical Investigators. Development of a simple noninvasive index to predict significant fibrosis in patients with HIV/HCV coinfection. Hepatology 2006;43:1317-25.
7. Wai C-T, Greenson JK, Fontana RJ, et al. A simple noninvasive index can predict both significant fibrosis and cirrhosis in patients with chronic hepatitis C. Hepatology 2003;38:518-526.
8. Kim WR, Therneau TM, Wiesner RH, et al. A revised natural history model for primary sclerosing cholangitis, In Mayo Clinic Proceedings, Elsevier, 2000.
9. Ewels P, Magnusson M, Lundin S, et al. MultiQC: summarize analysis results for multiple tools and samples in a single report. Bioinformatics 2016;32:3047-8.
10. Rognes T, Flouri T, Nichols B, et al. VSEARCH: a versatile open source tool for metagenomics. PeerJ 2016;4:e2584.
11. Bolyen E, Rideout JR, Dillon MR, et al. Reproducible, interactive, scalable and extensible microbiome data science using QIIME 2. Nat Biotechnol 2019;37:852-857.
12. Quast C, Pruesse E, Yilmaz P, et al. The SILVA ribosomal RNA gene database project: improved data processing and web-based tools. Nucleic Acids Res 2013;41:D590-6.
13. McMurdie PJ, Holmes S. phyloseq: an R package for reproducible interactive analysis and graphics of microbiome census data. PLoS One 2013;8:e61217.
14. Kuhn M. Building predictive models in R using the caret package. Journal of statistical software 2008;28:1-26.
15. Xu S, Zhan L, Tang W, et al. MicrobiotaProcess: A comprehensive R package for deep mining microbiome. Innovation (Camb) 2023;4:100388.
16. Koller M. robustlmm: An R Package for Robust Estimation of Linear Mixed-Effects Models. Journal of Statistical Software 2016;75:1-24.
17. Zhou H, He K, Chen J, et al. LinDA: linear models for differential abundance analysis of microbiome compositional data. Genome Biol 2022;23:95.
18. Mallick H, Rahnavard A, McIver LJ, et al. Multivariable association discovery in population-scale meta-omics studies. PLoS Comput Biol 2021;17:e1009442.
19. Friedman JH, Hastie T, Tibshirani R. Regularization paths for generalized linear models via coordinate descent. Journal of statistical software 2010;33:1-22.
20. Wright M, Ziegler A. Ranger: a fast implementation of random forests for high dimensional data in Cþþ and R. arXiv preprint arXiv:1508.04409 2015.
21. Hechenbichler K, Schliep K. Weighted k-nearest-neighbor techniques and ordinal classification. 2004.
22. Robin X, Turck N, Hainard A, et al. pROC: an open-source package for R and S+ to analyze and compare ROC curves. BMC bioinformatics 2011;12:1-8.
