# Supplementary material for: Geography-independent mucosal microbiota alterations in primary sclerosing cholangitis persist after liver transplantation
Source: JHEP Rep. 2025 Dec 22;8(4):101716. doi: 10.1016/j.jhepr.2025.101716 (PMC12972987; doi:10.1016/j.jhepr.2025.101716)
Supplement: Multimedia component 2 [file mmc2.pdf]

JOURNAL
OF HEPATOLOGY

Journal of Hepatology

CTAT methods

Tables for a "Complete, Transparent, Accurate and Timely account" (CTAT) are now mandatory for all revised submissions. The aim is to enhance the reproducibility of methods.

- Only include the parts relevant to your study
- Refer to the CTAT in the main text as 'Supplementary CTAT Table'
- Do not add subheadings
- Add as many rows as needed to include all information
- Only include one item per row

If the CTAT form is not relevant to your study, please outline the reasons why:

1.1 Antibodies

Name	Citation	Supplier	Cat no.	Clone no.
NA				

1.2 Cell lines

Name	Citation	Supplier	Cat no.	Passage no.	Authentication test method

1.3 Organisms

Name	Citation	Supplier	Strain	Sex	Age	Overall n number
NA						

1.4 Sequence based reagents

Name	Sequence	Supplier
NA		

1.5 Biological samples

Description	Source	Identifier
Intestinal tissue biopsy	taken during colonoscopy of healthy individuals or people with liver and/or intestinal disease before or after liver transplantation	

1.6 Deposited data

Name of repository	Identifier	Link

Sequence Read Archive database	PRJNA1250244	https://www.ncbi.nlm.nih.gov/sra

1.7 Software

Software name	Manufacturer	Version
R		v4.3.1

1.8 Other (e.g. drugs, proteins, vectors etc.)

NA		

1.9 Please provide the details of the corresponding methods author for the manuscript:

Monika Cahova, monika.cahova@ikem.cz

2.0 Please confirm for randomised controlled trials all versions of the clinical protocol are included in the submission. These will be published online as supplementary information.

NA
