# Supplementary material for: Geography-independent mucosal microbiota alterations in primary sclerosing cholangitis persist after liver transplantation
Source: JHEP Rep. 2025 Dec 22;8(4):101716. doi: 10.1016/j.jhepr.2025.101716 (PMC12972987; doi:10.1016/j.jhepr.2025.101716)
Supplement: Multimedia component 4 [file mmc4.pdf]

JHEP|Reports

Geography-independent mucosal microbiota alterations in primary sclerosing cholangitis persist after liver transplantation

Authors

Lukas Bajer, Petra Polakovicova, Marie Heczkova, …, Ondrej Fabian, Johannes R. Hov, Monika Cahova

Correspondence

monika.cahova@ikem.cz (M. Cahova).

Graphical abstract

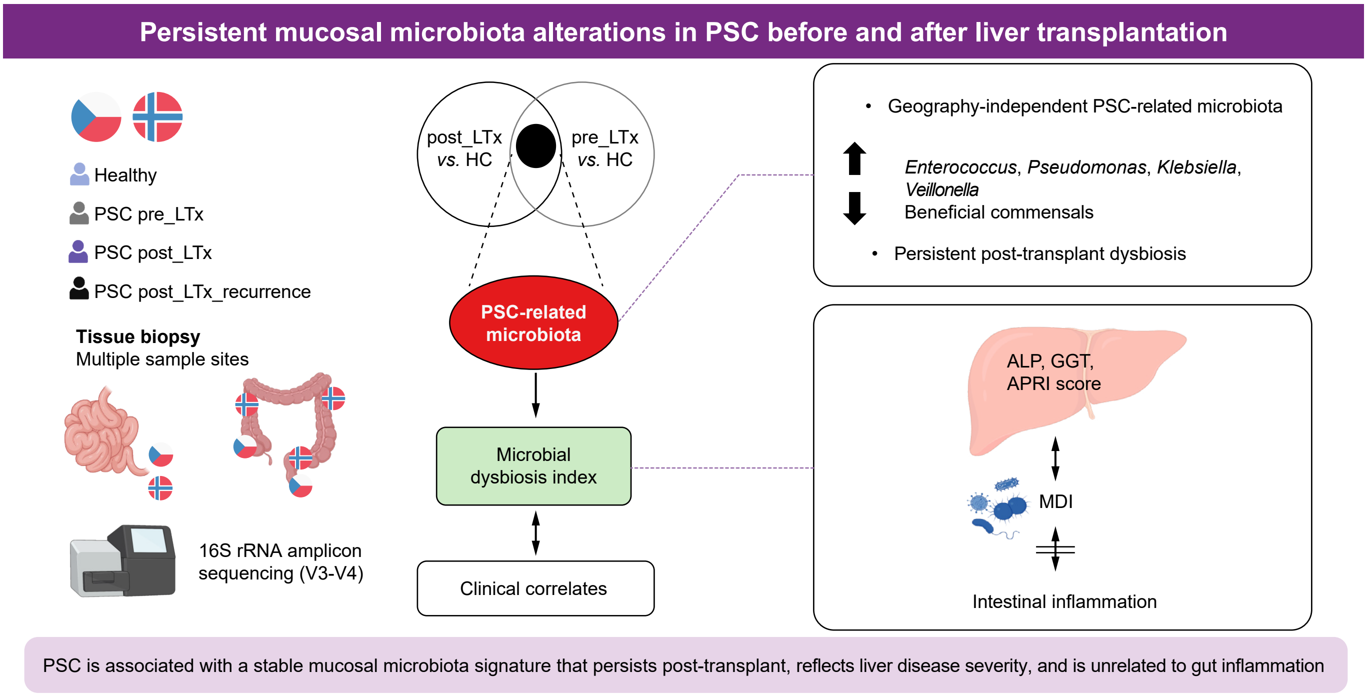

Highlights:

- PSC is associated with distinct, geography-independent mucosal microbiota alterations that persist after liver transplantation.
- Microbiota alterations correlate with liver disease severity but not with intestinal inflammation.
- PSC recurrence shows similar microbial trends to pre-transplant PSC but lacks a distinct microbiota profile.
- Targeting the microbiota may help treat PSC, prevent recurrence, and reduce post-transplant relapse.

Impact and implications:

This study provides an extensive evaluation of mucosa-associated microbiota in primary sclerosing cholangitis (PSC) before and after liver transplantation across two European cohorts. The persistence of PSC-related dysbiosis after transplantation highlights the importance of the gut–liver axis in PSC and supports further investigation into microbiota-driven mechanisms. Together with the strong association between microbiota composition and markers of cholestasis and fibrosis, this suggests potential clinical utility as an indicator of disease activity or even as a target for prevention or therapy.

https://doi.org/10.1016/j.jhepr.2025.101716

© 2025 The Author(s). Published by Elsevier B.V. on behalf of European Association for the Study of the Liver (EASL). This is an open access article under the CC BY license (http://creativecommons.org/licenses/by/4.0/). JHEP Reports, 2026, 8, 1–13

Geography-independent mucosal microbiota alterations in primary sclerosing cholangitis persist after liver transplantation

Lukas Bajer[1,2,†], **Petra Polakovicova**[3,4,†], Marie Heczkova[3], Kristian Holm[5,6,7], Mikal J. Hole[5,6,7], Mojmir Hlavaty[1,8], Alena Bohdanecka[3,8], Pavel Drastich[1], Filip Tichanek[9], Malin H. Meyer-Myklestad[10,11], Asle W. Medhus[6,12], Dag Henrik Reikvam[6,10], Kristin K. Jørgensen[5,6,13], Jan Brezina[1], Peter Macinga[1], Pavel Wohl[1], Ondrej Fabian[14], Johannes R. Hov[5,6,7,15,#], Monika Cahova[3,*,#]

JHEP Reports **2026**. vol. 8 | 1–13

Check for updates

Background & Aims: Primary sclerosing cholangitis (PSC)-associated alterations of fecal gut microbiota have already been described, but data on the mucosal microbiota are still limited. We aimed to further define disease-specific mucosal microbial patterns independent of geography and assess the relationship to liver transplantation (LTx), gut inflammation (inflammatory bowel disease), and PSC recurrence (rPSC).

Methods: We performed 16S ribosomal RNA gene (V3–V4) sequencing of ileocolonic biopsies from 115 patients with PSC (pre-LTx), 159 liver-transplanted patients (post_LTx, recurrence occurred in 38), and 96 healthy controls (HC) from Norway and the Czech Republic.

Results: Alpha diversity was lower in all PSC groups compared with HC. Elastic net models discriminated pre_LTx (AUC ileum 0.97; colon 0.93; p <0.001) and post_LTx PSC patients (AUC ileum 0.97; colon 0.97; p <0.001) from HC, and distinguished pre_LTx from post_LTx (AUC ileum 0.83; colon 0.83; p <0.001). The shared, cohort-independent PSC microbiota was dominated by *Enterococcus, Pseudomonas, Veillonella, Klebsiella*, and *Streptococcus*, while several common commensals were under-represented. A microbial dysbiosis index calculated from PSC-associated genera correlated negatively with alpha diversity and serum albumin, while a positive correlation was observed with markers of cholestatic disease (ALP, GGT) and liver fibrosis (APRI). There were no associations with the presence of inflammatory bowel disease or fecal calprotectin. Differences between post-LTx patients with and without recurrence were limited, but several genera deregulated in pre-LTx PSC (*Klebsiella, Bilophila, Coprococcus, Odoribacter*) showed similar trends in rPSC.

Conclusions: Our findings in two European countries revealed a distinct mucosal microbiota composition associated with PSC that persists after LTx. These microbial patterns correlate with the severity of liver injury in PSC but not with markers of intestinal inflammation.

© 2025 The Author(s). Published by Elsevier B.V. on behalf of European Association for the Study of the Liver (EASL). This is an open access article under the CC BY license (http://creativecommons.org/licenses/by/4.0/).

Introduction

Primary sclerosing cholangitis (PSC) is an immune-related chronic cholestatic liver disease affecting young adults and lacking effective medical therapy.[1] Although heritable, with multiple genetic risk factors identified, genetics explains only 10–20% of PSC liability,[2] indicating a major role for environmental exposures.

Up to 80% of patients with PSC have concomitant inflammatory bowel disease (IBD). This association has led to models implicating gut bacteria or their products in PSC pathogenesis,[3] supported by experimental data. While fecal microbiome studies consistently identify PSC-associated taxa, evidence from mucosal microbiota, potentially more relevant to pathogenesis,[4] remains limited and heterogeneous.[3] A key unresolved question is whether microbiota alterations contribute to PSC development or reflect secondary changes. Post-transplant patients may offer unique insights into this issue.[5]

PSC is a common indication for liver transplantation (LTx), and 15–30% of patients develop recurrent PSC (rPSC),[6] which increases the risk of graft loss or death.[7] Although the mechanisms of rPSC are poorly understood, they likely mirror those of PSC. The protective effect of colectomy before or during LTx[8] further suggests a microbiota-related component.

We have recently found in a single cohort of pretransplant (pre_LTx) and post-transplant (post_LTx) PSC patients from Norway[9] that there could be similar mucosal gut microbiota changes in pre_LTx PSC and post_LTx rPSC. In this study, we

* Corresponding author. Address: Institute for Clinical and Experimental Medicine, Videnska 1958, Prague, CR, Czech Republic. Tel.: +420 26136 5366.
E-mail address: monika.cahova@ikem.cz (M. Cahova).
† These authors have contributed equally to this work and share first authorship.
# These authors have contributed equally to this work and share the last authorship.
https://doi.org/10.1016/j.jhepr.2025.101716

aimed to define the mucosal microbiota in pre_LTx PSC and post_LTx rPSC, and assess how LTx affects microbiota normalization, by more than doubling the cohort with participants from the Czech Republic.

Patients and methods

Study population

This study includes two cohorts from the Czech Republic and Norway comprising 115 non-transplanted PSC (pre_LTx) patients, 159 transplanted PSC (post_LTx) patients, 50 patients transplanted for alcohol-related cirrhosis (ALD), and 96 healthy controls (HC). Both cohorts were collected consecutively as cross-sectional studies; therefore, no individual contributed samples both before and after LTx.

Czech PSC, ALD, and HC participants underwent colonoscopy at IKEM between 2021–2023. Norwegian patients with PSC were sampled during colonoscopy at Rikshospitalet, Oslo University Hospital (2005–2008), and Norwegian HC were individuals undergoing polyp surveillance (2016–2022); raw sequencing reads were available from a previous study.[9] PSC and IBD diagnoses followed clinical guidelines.[1,10] rPSC was diagnosed based on cholangiographic or histologic features consistent with PSC in the absence of defined secondary causes.[7,11]

Samples were collected from multiple gut sites: terminal ileum in both cohorts; cecum and rectum in the Czech cohort; and ascending, descending, and sigmoid colon in the Norwegian cohort. For comparisons and merging of datasets, all colon segments were analyzed under a unified "colon" category. A separate analysis of right *vs*. left colon was also performed by merging cecum + ascending colon (right) and descending colon + rectum (left). Further details are provided in Tables 1 and S1.

Sample collection, storage, library preparation, and sequencing

Mucosal biopsies were sampled using standard forceps. PSC samples from the Norwegian cohort were snap-frozen in dry tubes and stored without preservatives, while biopsies from HC were preserved in RNAlater (ThermoFisherScientific, Waltham, MA); Czech samples were preserved by DNA/RNA Shield (Zymoresearch, Irvine, California, USA). All samples were stored at −80 °C until analysis. DNA from mucosal biopsies in the Czech cohort was isolated by QIAmp PowerFecal DNA Kit (Qiagen, Hilden, Germany), while for Norwegian samples, All Prep DNA/RNA mini kit (Qiagen, Hilden, Germany) was used. PCR amplicons of the V3–V4 hypervariable regions of the bacterial 16S rRNA gene were sequenced on the Illumina MiSeq platform.

Bioinformatics processing

The Illumina paired-end reads were quality-checked, and after preprocessing, amplicon sequence variants (ASVs) were generated with Deblur in QIIME2 (2024.2) after trimming reads to 400 bp. The amplicon-region-specific Naive Bayes classifier was trained based on the SILVA Ref NR 99 database v 138.1 [Quast] via RESCRIPt QIIME 2 plugin.

Statistical analysis

All statistical analyses were conducted using R v4.3.1. The Czech and Norwegian cohorts were merged at the ASV level and then divided into two segments: terminal ileum and colon. As the first step, post_LTx, pre_LTx, and HC samples were analyzed. In the second step, the post_LTx group was further divided into rPSC and non-rPSC groups, with HC also being analyzed. An IBD *vs.* no-IBD comparison was performed within patients with PSC (pre_LTx and rPSC individuals combined). In each step, alpha diversity, beta diversity, and differential abundance analyses were conducted, and a binary classifier was trained to assess the discriminating power between groups. For all calculations, ASVs were aggregated to the genus level except for alpha diversity, which was calculated at the ASV level only. Where applicable, the Benjamini-Hochberg correction was applied to control the false discovery rate (FDR) for multiple comparisons.

Filtering: All samples with a read depth below 10,000 reads were removed. The nearZeroVar function from the caret package v6.0-94[12] with default parameters was used to filter low-prevalent and low-abundant taxa. This filtering step was applied to all analyses except for alpha diversity.

Alpha diversity (ASV Richness, Shannon index) was calculated on rarefied data (10,000 reads) at the ASV level using the MicrobiotaProcess package v1.14.1.[13] For the ileum, we employed a linear fixed-effects model that accounted for the effect of Cohort and its interaction with the Group. For the colon, a linear mixed-effects model was used, including Patient as a random effect.

Beta diversity was calculated as robust Aitchison distance using vegan package v2.6.4. Permutational ANOVA was performed to assess the effects of Group and Cohort. When the interaction effect was significant, *post hoc* analysis was conducted by separately testing the effect of the Group within each cohort and the effect of the Cohort within each group.

Differential abundance analysis was performed independently using two tools to minimize false positives, linDA from the MicrobiomeStat package v1.2 and the Maaslin2 package v1.16.0,[14] with filtered counts as input. The intersection of these two tools was used to identify differentially abundant taxa between groups. Taxa with a significant interaction effect were excluded based on individual *post hoc* analysis of the Czech and Norwegian cohorts. Only statistically significant taxa with log fold change that showed the same direction in both countries were retained.

Binary classification was performed using elastic net (ENET) with glmnet v4.1.8[15] and three supplementary models: random forest with ranger v0.17.0,[16] gradient boosting with gbm v2.2.2, and K-nearest neighbors with kknn v1.3.1.[17] Hyperparameter tuning was performed using five-fold cross-validation, with cv.glmnet for ENET parameters and the caret package for other models. Training and validation were performed via bootstrapping (n = 500) on clr-transformed data. In the colon dataset, bootstrapping was pseudo-randomized to keep each patient's samples in the same set (training or

Table 1. Patient characteristics.

	Pre_LTx		Post_LTx Non-rPSC		Post_LTx rPSC		Healthy	
	Norway[#]	Czech	Norway[#]	Czech	Norway[#]	Czech	Norway[#]	Czech
F/M [n]	19/65	12/19	11/27	26/57	4/9	3/22	8/32	30/26
Age [years]	40 (17; 77)	35 (17; 63)	48 (23; 70)	48 (32; 81)	51 (29; 64)	48 (26; 81)	62 (33; 94)	50 (23; 68)
IBD yes/no [n]	66/18	25/6	31/7	72/11	11/2	24/1	NA	NA
Time since Tx (years)	NA	NA	2.1 (0.5; 11.5)	7.4* (1.0; 26.7)	4.3 (1.1; 18.9)	6.7 (1.1; 20.4)	NA	NA
ATB 3 mo prior sampling (n)	—	11	—	16	—	8	—	0
Total bilirubin [µmol/L]	24 (3; 319)	62* (9;500)	19.5 (11; 49)	18 (5; 132)	24 (13; 52)	24 (6; 148)	—	21 (3; 68)
AST [µkat/L]	1.1 (0.2; 8.6)	1.6 (0.5; 4.0)	0.6 (0.2; 3.2)	0.4*** (0.2; 1.7)	1.1 (0.3, 4.0)	0.5 (0.2; 1.9)	—	0.5 (0.3; 1.9)
ALT [µkat/L]	1.6 (0.5; 8.6)	1.7 (0.5; 5.2)	0.6 (0.1,4.1)	0.5** (0.3; 4.0)	1.1 (0.2; 4.5)	0.5 (0.3; 3.5)	—	0.5 (0.3; 1.7)
ALP [µkat/L]	4.2 (0.9; 17.3)	6.0* (1.6; 17.8)	1.3 (0.6; 7.9)	1.4 (0.6; 3.5)	3.7 (0.5; 13.9)	2.3 (0.5; 13.1)	—	1.3 (0.7; 2.3)
GGT [µkat/L]	—	2.6 (0.6; 15.2)	—	0.4 (0.1; 4.8)	—	1.4 (0.3; 13.7)	—	0.4 (0.2; 5.0)
INR [-]	—	1.1 (0.9; 1.8)	—	1.1 (0.9; 1.6)	—	1.1 (1.0; 1.7)	—	1.1 (1.1; 1.2)
Creatinine [µmol/L]	65 (39; 232)	60 (44; 101)	84 (59; 159)	87 (49; 248)	81 (65; 133)	85 (50; 780)	—	77 (50; 115)
Albumin [G/L]	41 (26; 49)	38* (20; 50)	43 (35; 49)	45** (26; 51)	38 (34; 50)	42 (26; 50)	—	50 (43; 57)
Fecal calprotectin [µg/G]	59 (1; 2,844)	123* (6;4,513)	30 (1; 1,945)	275*** (6; 4,821)	55 (10; 832)	564* (14; 4,301)	—	—
NANCY_max [-]	—	2 (0;4)	—	2 (0; 4)	—	2 (0; 4)	NA	NA
eMayo [-]	—	1 (0; 2)	—	1 (0; 3)	—	1 (0; 2)	NA	NA
Mayo_DAI [-]	—	2 (0; 5)	—	1 (0; 8)	—	2 (0; 7)	NA	NA
Mayo_PSC risk score [-]	0.3 (2.2; 3.5)	0.3 (2.2; 3.5)	0.3 (1.1; 2.2)	—	1.0 (1.4; 3.1)	—	NA	NA
AOM_score [-]	1.9 (0.5; 3.8)	2.0 (1.1; 5.0)	1.9 (0.8; 2.7)	—	2.2 (1.2; 3.5)	—	NA	NA
APRI_score [-]	0.7 (0.1; 5.5)	1.1 (0.3; 14.3)	0.5 (0.2; 2.3)	0.3*** (0.1; 1.5)	0.7 (0.2; 3.6)	0.4* (0.1; 1.4)	NA	NA
FIB-4_score [-]	1.1 (0.8; 8.6)	1.6 (0.4; 23.1)	1.5 (0.6; 3.6)	1.1* (0.3; 5.0)	2.2 (0.5; 5.8)	1.1* (0.5; 3.1)	NA	NA
MELD_score [-]	—	8.2 (6.4; 18.0)	—	8.0 (6.4; 14.6)	—	7.8 (6.5; 22.8)	NA	NA
Platelets [10^9/L]	265 (37; 712)	201 (20; 477)	197 (101; 434)	208 (41; 442)	212 (69; 409)	213 (83; 416)	—	—

Data are given as median (min;max). Statistical differences between cohorts were evaluated using the Mann–Whitney *U* test. ALP, alkaline phosphatase; AOM, Amsterdam-Oxford model; APRI, aspartate aminotransferase-to-platelet ratio index; AST, aspartate aminotransferase; ATB 3 mo prior sampling, number of patients prescribed antibiotics during 3 months prior sampling; eMayo, endoscopic Mayo index; FIB-4, fibrosis-4 index; GGT, gamma-glutamyltransferase; IBD, inflammatory bowel disease; INR, international normalized ratio; Mayo_DAI, Mayo disease activity index; MELD, model for end-stage liver disease; NA, not applicable.

*p <0.05; **p <0.01; ***p <0.001, statistically significant difference Norwegian *vs.* Czech cohort within respective group.

[#]These patients were already described in the previous study.[9]

validation). Model performance metrics expressed as AUC were calculated based on an out-of-bag principle using pROC package v 1.18.5.[18] Optimism-corrected AUC was estimated as the mean AUC from the validation performance across the bootstrapped samples. To prevent training errors and overfitting, we validated all models by shuffling sample labels, confirming their poor performance with an AUC not exceeding 0.57.

Microbial dysbiosis index (MDI) was defined as the ratio of the total abundance of taxa increased in PSC to the abundance of taxa decreased in PSC. We modified the previously described approach,[19] calculating MDI as the log of [total abundance in organisms increased in disease] over [total abundance of organisms decreased in disease]. Since we used clr-transformed data, we calculated this index as the difference between the two values. MDI was calculated separately for ileum and colon samples at the genus level.

Spearman's correlation coefficient was used to assess the relationship between the MDI and clinical parameters. For the colon samples, the correlation was computed 100 times, with each calculation performed on a randomly selected sample from each patient. The final reported correlation represents the average across all iterations. A correlation was considered significant if at least 90 out of the 100 iterations yielded a $p < 0.05$.

A detailed description of the methodology is provided in the supplementary methods.

Results

General characterization of mucosal microbiota

Data from two independent cohorts of patients from Norway and the Czech Republic were included. Patient characteristics are listed in Tables 1 and S1. A total of 1,042 samples from 366 participants were retained after filtering, *i.e.* after excluding samples below 10,000 reads, and further analyzed. In 281 samples from the terminal ileum, after filtering, 164 genera remained, belonging to 10 phyla. In 761 samples from the colon, representing multiple sampling sites, after filtering, 126 genera remained, representing eight phyla (Tables S2 and 3). The workflow of further analysis is shown in Fig. 1.

PSC is associated with lower mucosal microbiota diversity

Both pre_LTx and post_LTx PSC patients exhibited significantly lower mucosal microbiota alpha diversity in both the terminal ileum and colon compared with HC, as measured by ASV Richness. A reduction in the Shannon diversity index was also observed, with the most pronounced decrease in pre_LTx patients. In post_LTx patients, the Shannon index remained lower than in HC only in the colon, albeit it did not reach statistical significance ($p = 0.06$). The differences between the pre_LTx and post_LTx groups were minor, with a trend toward lower alpha diversity in pre_LTx patients, primarily influenced by the Czech cohort (Fig. 2A, Table S4).

PSC is associated with distinct mucosal microbiota composition

Considering the global microbiota composition (Fig. 2B), all three investigated groups overlapped in the two-dimensional space. Despite the large overlap, HC samples overall were distinct from the pre_LTx and post_LTx groups (permutational ANOVA, $p < 0.05$ in all comparisons, Table S5). The effect of the cohort was also significant in all cases. These results were consistent across datasets from both the terminal ileum and colon. The interaction between disease group and cohort was significant when analyzing the differences between pre_LTx and HC and between pre_LTx and post_LTx in the terminal ileum. *Post hoc* analyses revealed that the pre_LTx and HC groups were significantly different in both cohorts, while significant beta diversity differences between pre_LTx and post_LTx were observed only in the Norwegian cohort.

We further examined whether individual microbiome compositions could accurately predict group membership using a machine learning (ML) approach. ENET model results are shown in Fig. 2C. High AUC values were achieved when comparing pre_LTx *vs.* HC (terminal ileum: 0.97, 0.92-0.99, $p < 0.001$; colon: 0.93, 0.84-0.99, $p < 0.001$) or post_LTx *vs.* HC (terminal ileum: 0.83, 0.75-0.92, $p < 0.001$; colon: 0.97, 0.94-0.99, $p < 0.001$). Values in brackets represent the mean, 95% CI, and p value. The model also exhibited moderate discriminatory ability for distinguishing between pre_LTx and post_LTx (AUC in terminal ileum: 0.83, 0.75-0.92, $p < 0.001$; colon: 0.83, 0.74-0.91, $p < 0.001$). To ensure that the model was not driven only by one of the cohorts, we also built the models separately on the Czech and Norwegian datasets, obtaining stable and comparable performances. Similar outcomes were obtained using three other ML models, *i.e.* random forest, K-nearest neighbor, or gradient boosting (Table S6).

Next, we performed a differential abundance analysis in merged Czech and Norwegian datasets. First, we compared the taxonomical composition of pre_LTx *vs.* HC and post_LTx *vs.* HC. The former comparison reflects the effect of the disease itself, potentially together with disease stage effects, while the latter combines the effect of disease and transplantation, immune suppressive therapy, etc. The intersection of these two sets could be considered the specific PSC-associated microbiota (Fig. 1). According to this approach, the PSC-associated microbiota in the terminal ileum included 15 genera (Fig. 3A, Table S7). Two of them, *Enterococcus and Pseudomonas*, were overrepresented in PSC, while 13, including *Parabacteroides, Oscillibacter, Odoribacter, Lachnospiraceae_FSC020_group, Lachnoclostridium, Holdemania, Fusicatenibacter, Faecalibacterium, Enterorhabdus, Coprococcus, Butyricimonas, Barnesiella, Alistipes*, were more abundant in HC. In the colon, 22 genera were associated with PSC (Fig. 3B, Table S8). Genera *Veillonella, Pseudomonas, Klebsiella, Hungatella, Rothia, Dialister,* and *Enterococcus* were more abundant in patients with PSC, while 15 genera were less abundant, with extensive overlap with the findings in the terminal ileum.

In the Czech cohort, mucosa-associated microbiota data were available for patients who underwent LTx due to ALD, providing a control group distinct from healthy individuals (Table S9). To determine whether the identified PSC-associated microbiota signature is disease-specific or reflective of liver disease more broadly, we conducted a nested analysis limited to Czech post_LTx ALD patients. By comparing the taxonomic composition of these patients with HC, we defined the "Czech" ALD-associated microbial signature. This was then compared with the geographically independent PSC-associated taxa identified previously.

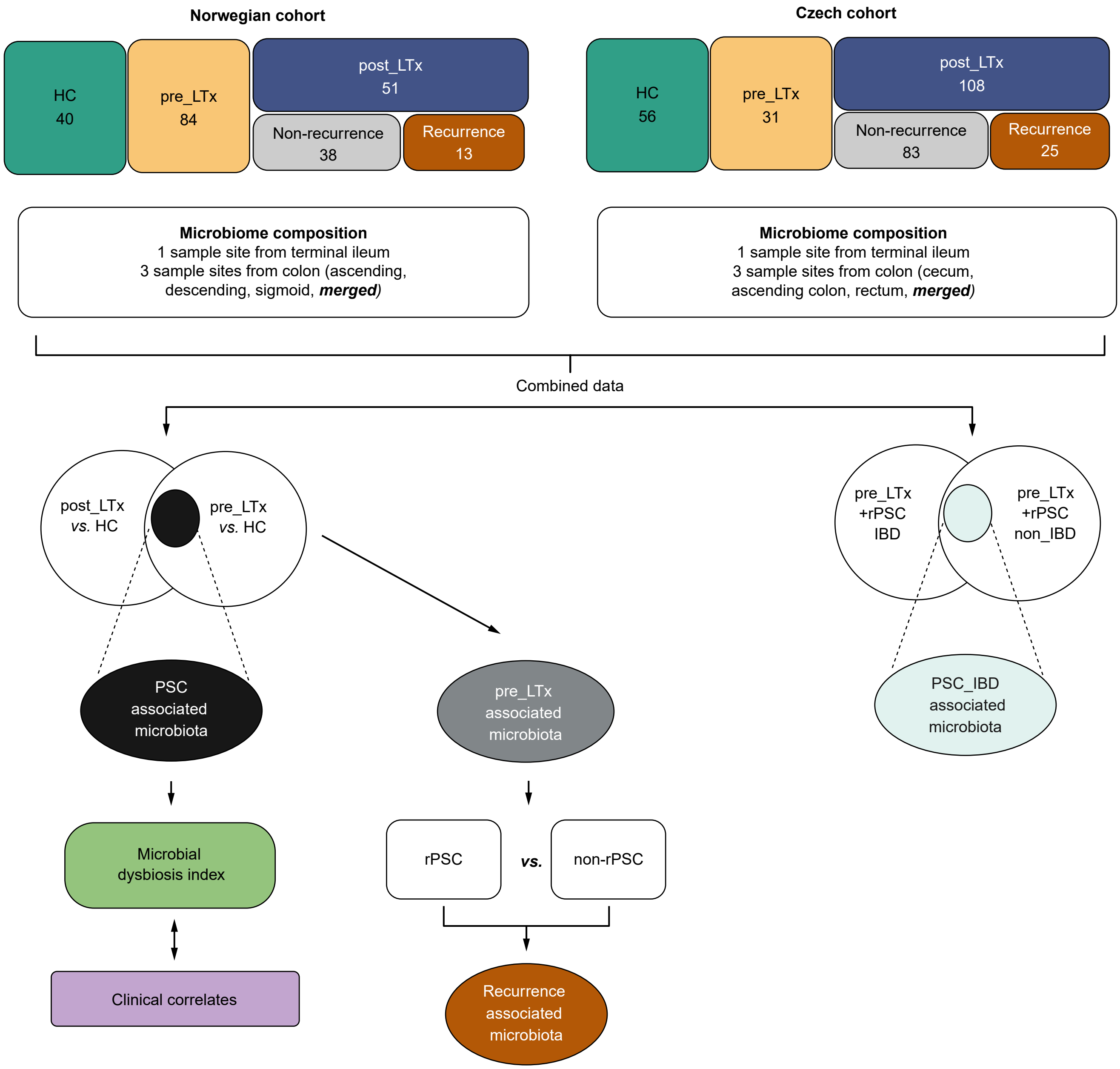

Fig. 1. Design of the study. Mucosal microbiota composition was analyzed in Czech and Norwegian cohorts, followed by statistical evaluation including predictive modeling, alpha diversity, and differential abundance analyses. Taxa selected in differential abundance tests were used to define PSC-related, recurrence-related, and PSC_IBD-related signatures. A microbial dysbiosis index based on PSC-related taxa was then calculated to explore clinical associations. HC, healthy controls; IBD, inflammatory bowel disease; LTx, liver transplantation; MDI, microbial dysbiosis index; PSC, primary sclerosing cholangitis; rPSC, recurrent primary sclerosing cholangitis.

Based on this comparison, nine bacterial taxa (*Enterorhabdus, Barnesiella, Butyricimonas, Parabacteroides, Holdemania, Fusicatenibacter, Lachnoclostridium, Lachnospiraceae_FCS020_group, Enterococcus*) in the terminal ileum and six (*Rothia, Parabacteroides, Fusicatenibacter, Lachnoclostridium, Colidextribacter, Dialister, Klebsiella*) in the colon were specific to the PSC-associated signature. This result further supports the existence of a PSC-related microbial signature. However, given that the analysis could only be performed on a limited set of cases and may be cohort-biased, we continued with further studies according to the procedure shown in Fig. 1, working with the full list of identified PSC-associated taxa.

Mucosal microbiota and PSC recurrence

Next, we assessed the relationship between gut microbiota composition and disease recurrence post_LTx. Alpha diversity analysis revealed no significant difference between rPSC and non-rPSC, although both groups had lower alpha diversity

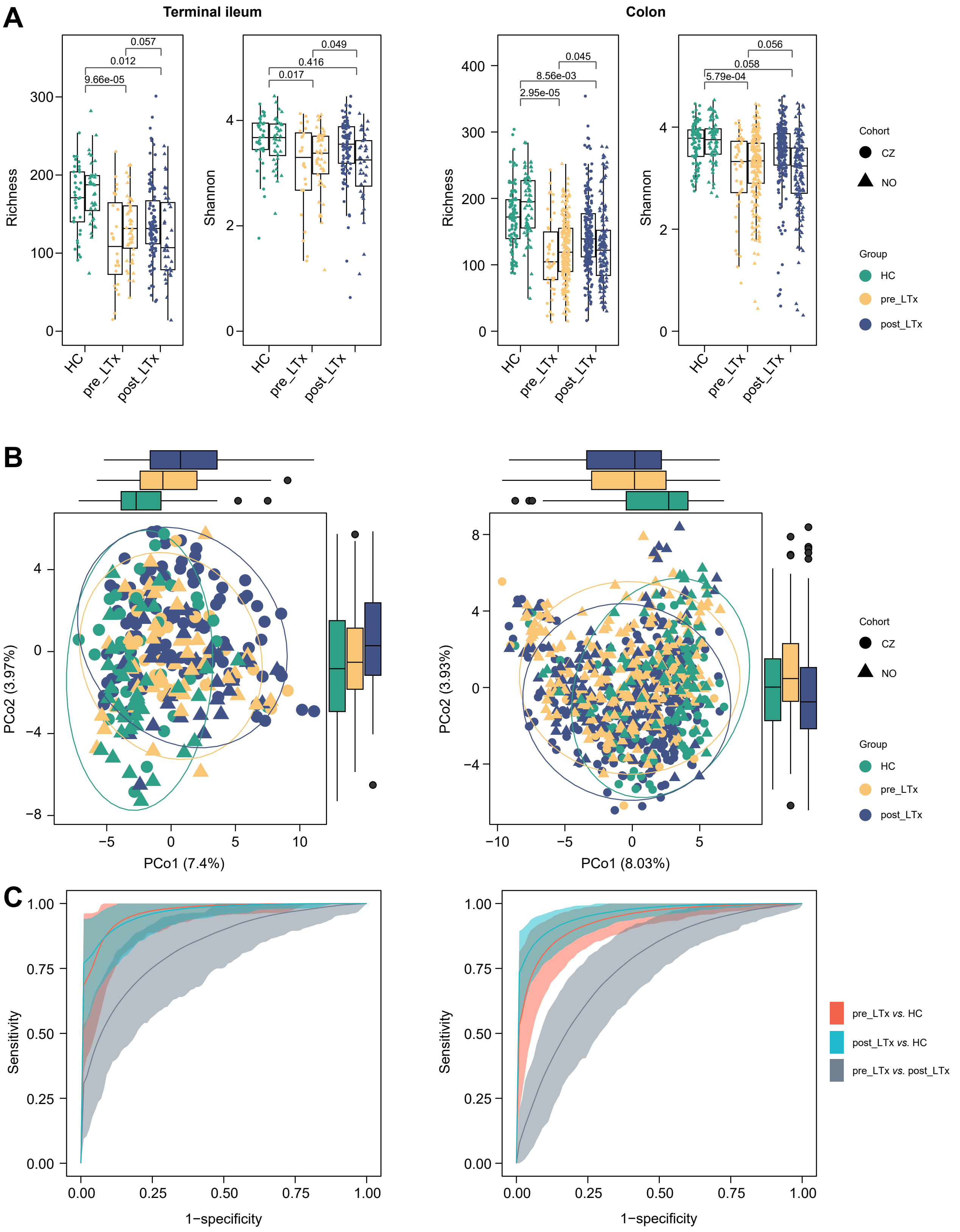

Fig. 2. Microbiota composition in pre_LTx, post_LTx, and HC groups. (A) Alpha diversity (Richness, Shannon) in the terminal ileum and colon based on ASVs; between-group differences were assessed in merged cohorts (ileum: linear fixed-effects model; colon: linear-mixed effect model, BH correction). Grey values indicate significant interaction effects with inconsistent *post hoc* results. (B) PCoA plots showing distinct microbiota composition across groups; explained variance is shown in brackets. (C) Performance of elastic net predictive models (ROC curves with 95% CI). HC, healthy controls; LTx, liver transplantation; PCo, principal coordinate.

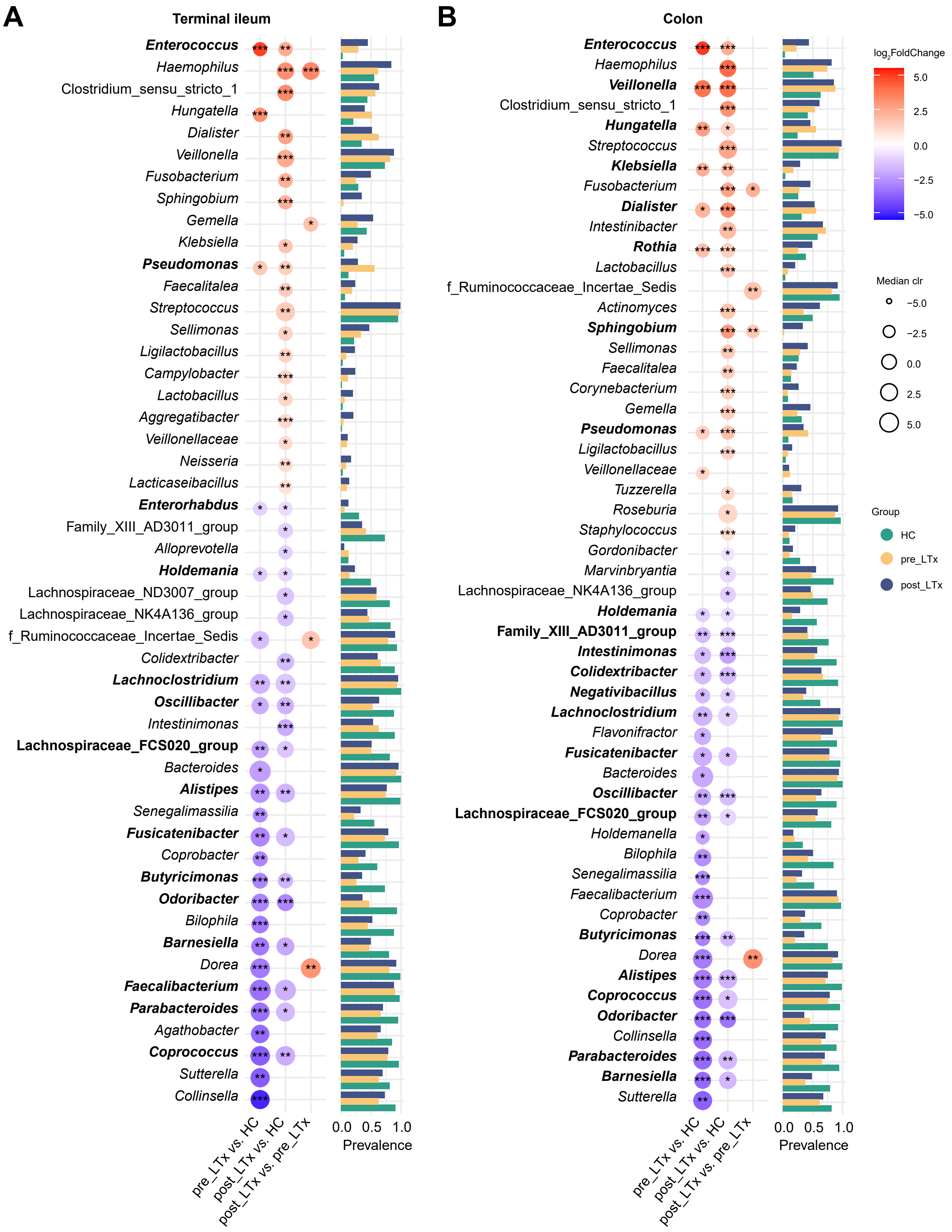

Fig. 3. Comparison of the relative abundance of bacterial taxa: pre_LTx, post_LTx, and HC groups. (A) Terminal ileum; (B) Colon. PSC-related taxa were identified as those differing from HC in both pre_LTx and post_LTx groups (bold). Prevalence refers to the proportion of samples in which a taxon is detected. *q <0.05; **q <0.01; ***q <0.001 (linDA). HC, healthy controls; LTx, liver transplantation; PSC, primary sclerosing cholangitis.

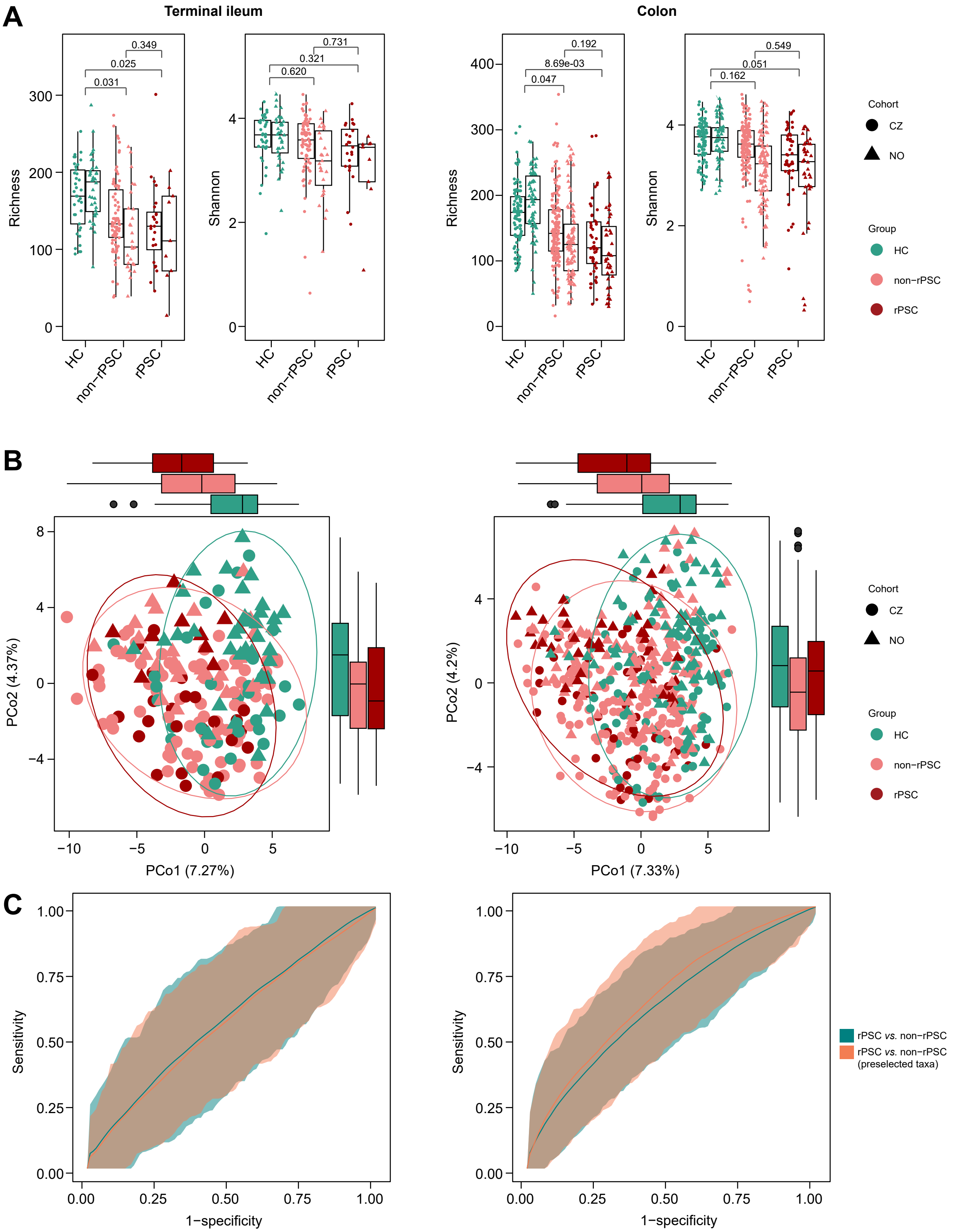

Fig. 4. Microbiota composition in post_LTx_non-rPSC, post_LTx_rPSC, and HC groups. (A) Alpha diversity (Richness, Shannon) in the terminal ileum and colon based on merged cohorts; grey values mark significant interaction effects with inconsistent *post hoc* outcomes (ileum: linear fixed-effects model; colon: linear-mixed effect model, BH correction) (B) PCoA plots showing group-level differences; explained variance in brackets. (C) ROC curves for elastic net predictive models. HC, healthy controls; LTx, liver transplantation; PCo, principal coordinate; PSC, primary sclerosing cholangitis; rPSC, recurrent primary sclerosing cholangitis.

determined as ASV Richness than healthy controls (Fig. 4A, Table S4). There was also no statistically significant difference in the overall microbiota composition between the rPSC and non-rPSC groups (Fig. 4B, Table S5). The predictive models confirmed the high discriminatory power of individual microbiota to distinguish non-rPSC and rPSC from HC, but not rPSC from non-rPSC (Fig. 4C, Table S6).

In line with our previous study,[9] we wanted to test specifically the genera discriminating between pre_LTx_PSC and HC in recurrent PSC. All genera with different relative abundance in PSC *vs.* HC at Q_{FDR} <0.05 were compared between post_LTx_non-rPSC and post_LTx_rPSC. Even within this subset of bacteria, there were no significant differences between the groups following FDR correction (Fig. S1). However, there were trends towards numerical increases of *Klebsiella* in post_LTx_rPSC (colon) and a reduction of *Bilophila* (colon and terminal ileum), *Odoribacter* and *Coprococcus* (only terminal ileum), as indicated by unadjusted *p* values (Tables S7 and S8). The performance of the ENET model trained on the pre_LTx_PSC-associated subset of genera was poor in the terminal ileum (AUC: 0.56, 0.39-0.72; p = 0.360). Performance in the colon was slightly improved (AUC: 0.66, 0.50–0.79; p = 0.051) compared to the terminal ileum; however, wide confidence intervals still reflect high uncertainty in model performance (Fig. 4C, Table S6).

PSC microbiota does not exhibit location-related associations in the colon

Regional differences in mucosal microbiota composition could be of particular interest, given that PSC with IBD may display an atypical colonic distribution that remains unexplained (*e.g.* right-sided dominance, backwash ileitis, rectal sparing). To assess potential location-related effects, we performed separate analyses of samples from the right (cecum, ascending colon) and left (descending colon, rectum) sides of the colon. Alpha and beta diversity measures, as well as the outcomes of predictive models, were comparable between the two regions. Therefore, the results obtained on the merged dataset were not driven by one location only and could be attributed to the entire mucosal colonic microbiota (Tables S10–12).

Microbial dysbiosis index positively correlates with cholestasis markers

For clinical associations, we calculated a MDI based on PSC-associated microbial taxa per segment. Numerically, the MDI was highest in rPSC, followed by non-rPSC and pre_LTX, irrespective of the segment (Fig. 5A). MDI was strongly and inversely correlated with alpha diversity (Fig. 5B). MDI correlated positively with markers of cholestatic liver disease, alkaline phosphatase (ALP) and gamma-glutamyltransferase (GGT), and with the fibrosis score APRI (aspartate aminotransferase-to-platelet ratio index), and negatively with serum albumin (Fig. 6A).

We also examined which bacteria within the MDI contributed to the clinical correlations. In the terminal ileum, 7 out of 15 PSC-associated genera were correlated with at least one of the previously mentioned clinical parameters. *Enterococcus*, which was increased in PSC, showed a trend of positive correlations with markers of cholestasis (ALP and GGT) and, together with *Pseudomonas,* negative correlations with serum albumin. Genera decreased in PSC samples exhibited opposite trends. A similar pattern was observed in the colon, where 17 out of 22 PSC-associated genera correlated with at least one of the parameters (Fig. 6B,C).

Intestinal inflammation, gut microbiota, and PSC markers

Microbial composition analysis revealed no significant differences in alpha diversity or beta diversity between patients with PSC with and without IBD (Fig. S2A and C). Using the MaAsLin2 tool, we observed significantly decreased abundance of *Akkermansia* in both the terminal ileum and colon in patients with PSC and IBD compared to those with PSC without IBD. In contrast, the Linda tool did not find any difference between these two subsets (Fig. S3). Using a fecal calprotectin cut-off of 250 μg/g, with values below considered remission, we observed no microbiota differences between low- and high-inflammation groups in the terminal ileum or colon (Fig. S2B and D). Also, intestinal inflammation as measured by fecal calprotectin did not correlate with ALP or GGT.

Discussion

Here, we report a cross-sectional investigation of the mucosal microbiota of the ileocolon of 115 non-transplanted and 172 transplanted patients with PSC and 96 HCs from Norway and the Czech Republic, representing a significant step forward in sample size and analytic depth. The major findings can be summarized as follows: 1) we confirmed PSC-related microbiota features in the terminal ileum and colon across two geographically distinct populations; 2) gut microbiota composition had high discriminative power between HC and patients with PSC, irrespective of transplantation status, suggesting that PSC-related microbiota persist after LTx; 3) post-transplant, the gut microbiota in rPSC and non-rPSC was similar, but with some parallel trends between rPSC and pre_LTx_PSC; and 4) there was no association between IBD or intestinal inflammation and the gut microbiota in PSC, despite a trend toward lower *Akkermansia muciniphila* in patients with concomitant IBD.

The mucosal microbial diversity was reduced in the colon and terminal ileum in PSC, both before and after LTx. This expands on and confirms our previous work from the Norwegian subset only, by doubling the study size with a Czech cohort, allowing us to establish this phenomenon independent of geography. Other earlier studies investigating the mucosal microbiota composition in PSC have been small (n = 10-31) and with inconsistent methodology, making it difficult to compare.[20–23] Fecal microbiota-based studies confirmed reduced microbial diversity in PSC,[24–26] and similar patterns are observed for individual taxa. We *found Enterococcus, Veillonella, Klebsiella,* and *Rothia* to be significantly more abundant in PSC than in HC, consistent with findings from stool studies.[24–26] Our results suggest that, despite regional differences, consistent PSC-associated features of the gut microbiota largely overlap in mucosal and fecal samples.

The gut microbiota composition in the post_LTx PSC population was markedly different from that in HC, but similar to that in pre_LTx PSC. This finding aligns with our previous results in the Norwegian subset.[9] Although comparative data are limited, these observations provide a contrast to liver disease of other etiologies, which are also commonly associated

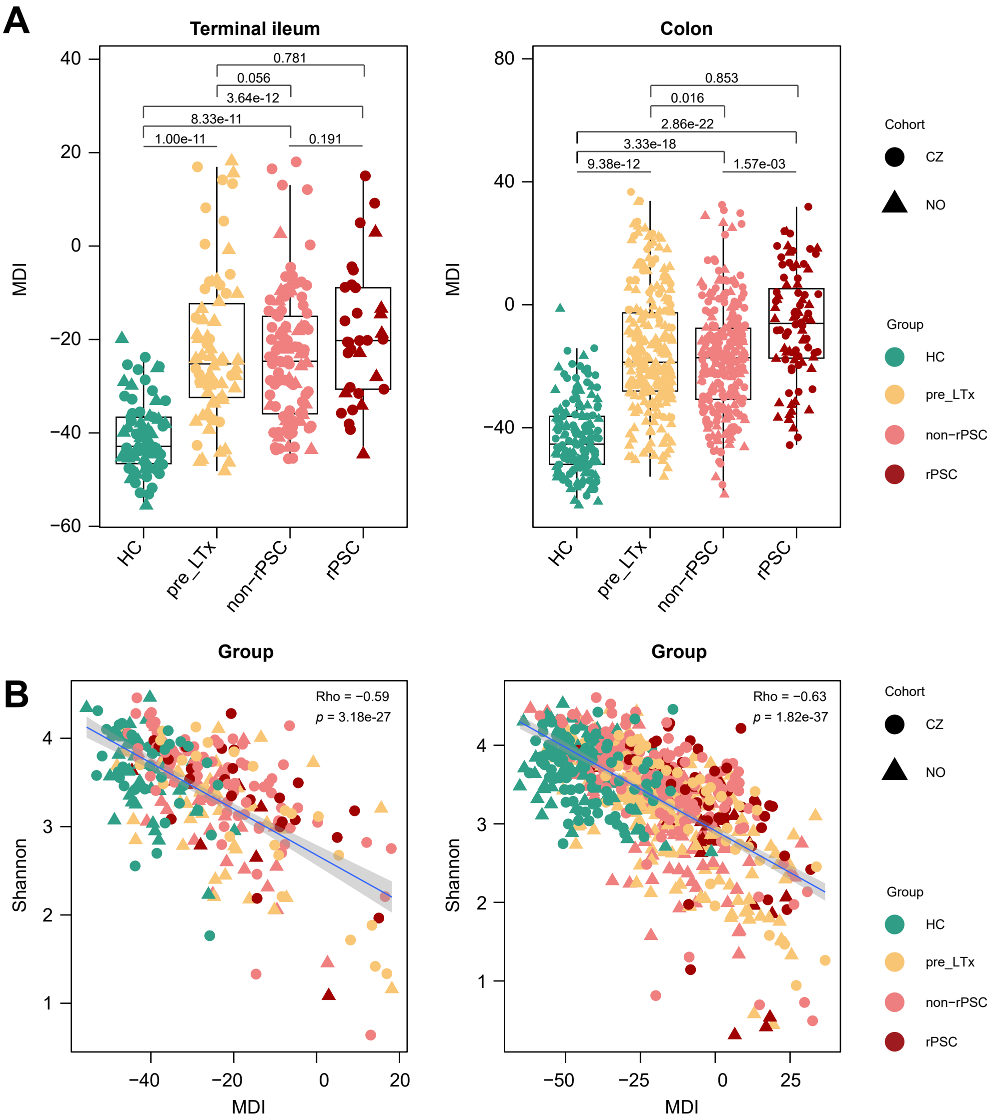

Fig. 5. MDI. (A) MDI in the terminal ileum and colon (ileum: linear fixed-effects model; colon: linear-mixed effect model, BH correction). (B) Relationship between MDI and alpha diversity (Shannon) (Spearman's test). HC, healthy controls; LTx, liver transplantation; MDI, microbial dysbiosis index; PCo, principal coordinate; PSC, primary sclerosing cholangitis; rPSC, recurrent primary sclerosing cholangitis.

with alterations in gut microbiota composition.[3,27–29] The altered function of the diseased liver, *e.g.* changes in bile acid metabolism, likely contributes to shaping the intestinal environment. In patients transplanted for hepatitis C, ALD, metabolic dysfunction–associated steatohepatitis, or hepatocellular carcinoma, significant post_LTx changes in gut microbiota have been reported alongside improved liver function. These changes include increased alpha diversity, enrichment of autochthonous taxa, and a reduction in potentially pathogenic taxa.[30–32] In contrast, in PSC the post_LTx microbiota showed deregulation of many taxa already altered pre_LTx, often in the same direction and in some cases more pronounced after transplantation. While this study does not follow the trajectory of the gut microbiota composition during transplantation, but instead compares transplanted and non-transplanted patients, our data indicate that at the group level, LTx is insufficient for the recovery of gut microbiota to a near-healthy composition in PSC. Potential drivers include advanced surgery, hospitalization, chronic immunosuppression, and Roux-en-Y anatomy. Still, our observations raise the question of whether the PSC-associated microbiota could appear before disease onset.

Approximately 15-30% of patients transplanted for PSC develop a recurrence of the disease, which is associated with reduced survival.[6] The extensive PSC microbiota alterations persisting after transplant suggest that the gut could be a source of recurrence-driving factors. However, our data showed limited differences between rPSC and non-rPSC patients. Still, by hypothesizing that gut microbiota alterations in rPSC would mirror PSC without transplant, we found that several genera associated with pre_LTx PSC were also associated with rPSC. The findings included increased *Klebsiella* and reduced *Bilophila* and *Coprococcus*. Furthermore, an

ENET model of pre_LTx PSC-related colonic taxa did show some ability to separate rPSC from non-rPSC.

One important question is how microbiota alterations in PSC relate to disease stage, the degree of cholestasis, or intestinal inflammation. Across all samples, the modified MDI demonstrated a strong positive correlation with markers of cholestatic disease severity (ALP, GGT) and liver fibrosis (APRI score). Notably, we calculated a modified MDI compared with the original strategy by Gevers *et al.*,[19] which means that the associations cannot be directly compared across different studies. Considering the individual genera contributing to the index, multiple associations were found with markers of cholestasis, including a positive correlation with *Enterococcus*, a central finding in a previous study of PSC and IBD phenotypes.[33] The same study also found multiple associations with intestinal inflammation, measured by fecal calprotectin. This contrasts with our findings, which showed no differences in microbiota composition between PSC patients with and without IBD, despite a trend toward lower abundance in PSC-IBD, no stratification by fecal calprotectin, and no association between fecal calprotectin and the MDI. This may be considered surprising given the strong relationship between IBD and gut microbiota. However, this finding aligns with previous microbiota studies in PSC.[21–24] Recently, Wittek *et al.* reported that signs of IBD on the molecular level were present in all patients with PSC, even in the absence of clinically manifest IBD.[34] This observation suggests that PSC is inherently associated with intestinal inflammation, making it difficult to distinguish between PSC-IBD- and PSC-non-IBD-associated microbiota. _

A cross-sectional design cannot address causality, but our data strengthen the rationale for further studies to delineate mechanisms by which individual bacteria may cause or modify PSC. *Enterococcus* has consistently been found to have increased relative abundance in the PSC gut and in bile, and *Enterococcus* culture-positive bile is associated with worse outcomes.[35,36] At the molecular level, some fecal *Enterococci* may express the virulence factor cytolysin, which is linked to worse outcomes.[37] Some strains of *Klebsiella pneumoniae* may translocate across the gut barrier and induce or worsen biliary inflammation,[38] and *Klebsiella* is associated with poor survival in PSC.[9,39] *Veillonella* is increased in many liver diseases and could be a secondary phenomenon that thrives in this niche. However, there is also some evidence that it could influence the disease, *e.g.* via epithelial barrier dysfunction.[35,38,40] The spectrum of taxa reduced in PSC samples is more numerous and diverse, consisting of general commensals and potentially beneficial microbes. Taken together, the PSC microbiome is characterized by the overrepresentation of a few pathogenic taxa, which could have negative disease-modifying effects, combined with the depletion of protective commensal microbiota.

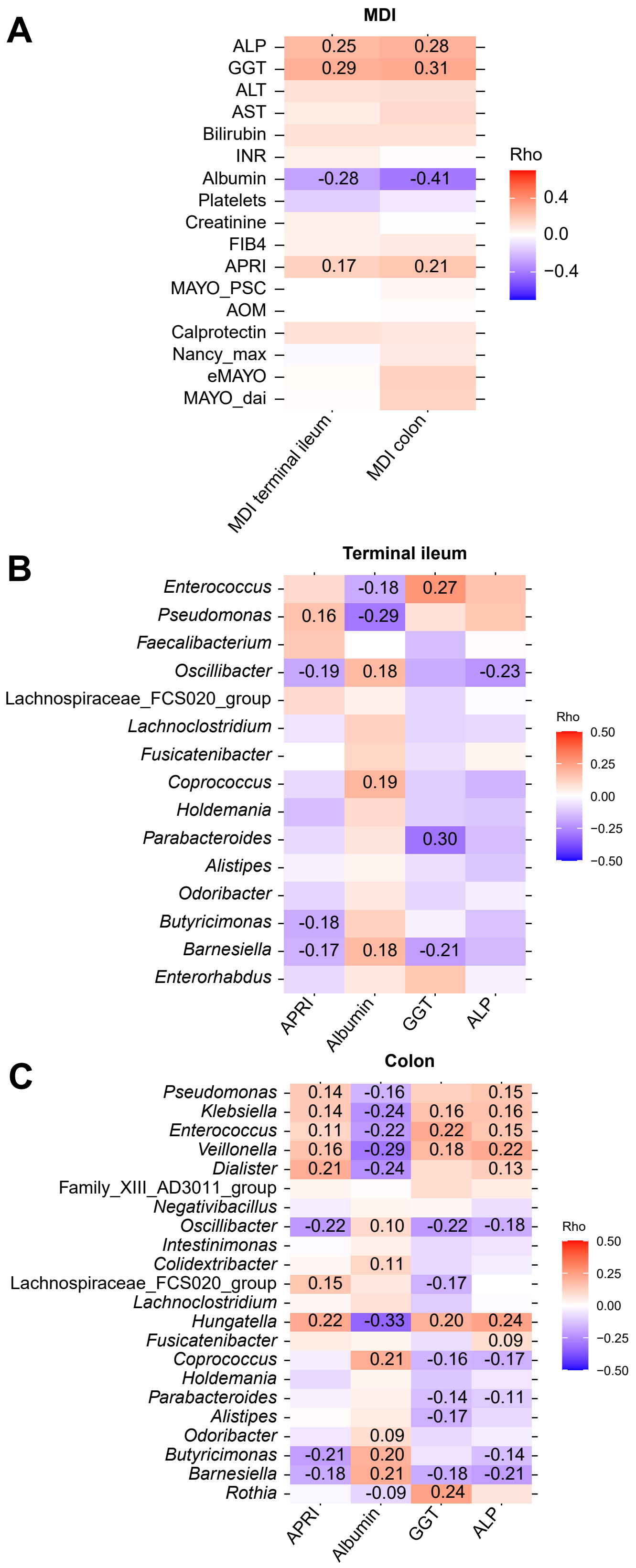

Fig. 6. Associations between MDI taxa and clinical parameters. (A) Correlations between MDI and selected clinical variables. (B) Correlations of individual MDI taxa with clinical outcomes in the terminal ileum. (C) Correlations of individual MDI taxa with clinical outcomes in the colon. Values show Spearman correlation coefficients; unannotated tiles indicate non-significant values (q >0.05). ALP, alkaline phosphatase; AOM, Amsterdam-Oxford model; APRI, aspartate aminotransferase-to-platelet ratio index; AST, aspartate aminotransferase; eMayo, endoscopic Mayo index; FIB-4, fibrosis-4 index; GGT, gamma-glutamyltransferase; IBD, inflammatory bowel disease; INR, international normalized ratio; Mayo_DAI, Mayo disease activity index; MDI, microbial dysbiosis index.

The key strength of this study lies in its focus on mucosal microbiota in two independent groups from geographically distinct locations. By combining new and previous[9] datasets using a completely different statistical approach designed explicitly for microbiota studies, we can present the most extensive mucosal microbiota study in PSC and show robust and geography-spanning alterations. However, several limitations should be acknowledged. We used 16S rRNA sequencing because shotgun metagenomics is not cost-effective in samples dominated by human DNA, although this approach has known drawbacks, including amplification bias and lower taxonomic resolution. Pre-analytical differences between cohorts*, i.e.* sample preservation media and DNA isolation, represent an additional source of variability. Although "country" was included as a covariate, it is unclear whether it captures only geography or also differences in sample handling. Moreover, detailed information on recent antibiotic use was unavailable for the Norwegian cohort. A disease control group was available only for post_LTx ALD patients in the Czech cohort, limiting our ability to distinguish disease-from transplantation-related effects and to determine whether the same pattern applies to the Norwegian cohort. Because the number of non-IBD patients was small, the IBD *vs.* no-IBD comparison required the pre_LTx and post_LTx rPSC subgroups to be merged, which may introduce residual confounding. Finally, the cross-sectional design precludes firm conclusions about links between dysbiosis and clinical outcomes. A prospective longitudinal study in patients with PSC after LTx is currently underway to address this limitation.

To conclude, a cross-sectional binational study on Norwegian and Czech patients with PSC identified disease-specific and geography-independent mucosal microbial alterations congruent with previously described findings. Parallel investigation of pre-transplant and post-transplant patients revealed that PSC-related dysbiosis remains despite resolution of the liver disease. Although we did not identify a specific microbial signature associated with recurrent PSC, the persisting dysbiosis after LTx might represent one of the risk factors contributing to disease recurrence.

Affiliations

[1]*Institute for Clinical and Experimental Medicine, Department of Hepatogastroenterology, Prague, CR, Czech Republic;* [2]*Department of Internal Medicine, 2nd Faculty of Medicine, Charles University, Prague, CR, Czech Republic;* [3]*Institute for Clinical and Experimental Medicine, Center for Experimental Medicine, Prague, CR, Czech Republic;* [4]*Faculty of Science, Charles University, Prague, CR, Czech Republic;* [5]*Norwegian PSC Research Center, Department of Transplantation Medicine, Oslo University Hospital, Oslo, Norway;* [6]*Institute of Clinical Medicine, University of Oslo, Oslo, Norway;* [7]*Research Institute of Internal Medicine, Oslo University Hospital, Oslo, Norway;* [8]*First Faculty of Medicine, Charles University, Prague, CR, Czech Republic;* [9]*Institute for Clinical and Experimental Medicine, Department of Data Science, Prague, CR, Czech Republic;* [10]*Department of Infectious Diseases, Division of Medicine, Oslo University Hospital, Oslo, Norway;* [11]*Department of Microbiology, Division of Laboratory Medicine, Oslo University Hospital, Oslo, Norway;* [12]*Department of Gastroenterology, Division of Medicine, Oslo University Hospital, Oslo, Norway;* [13]*Department of Gastroenterology, Akershus University Hospital, Lørenskog, Norway;* [14]*Institute for Clinical and Experimental Medicine, Department of Pathology, Prague, CR, Czech Republic;* [15]*Section of Gastroenterology, Department of Transplantation Medicine, Oslo University Hospital, Oslo, Norway*

Abbreviations

ALD, alcohol-related cirrhosis; ALP, alkaline phosphatase; ASV, amplicon sequence variant; ENET, elastic net; FDR, false discovery rate; GGT, gamma-glutamyltransferase; HC, healthy controls; IBD, inflammatory bowel disease; LTx, liver transplantation; MDI, microbial dysbiosis index; ML, machine learning; PSC, primary sclerosing cholangitis; rPSC, recurrent primary sclerosing cholangitis.

Financial support

This study was supported by MH CR in cooperation with the Czech Health Research Council under project No. NU21J-06-00027, by the project National Institute for Research of Metabolic and Cardiovascular Diseases (Programme EXCELES, Project No. LX22NPO5104) - Funded by the European Union - Next Generation EU and by MH CR – conceptual development of research organization (Institute for Clinical and Experimental Medicine – IKEM, IN 00023001). JRH was funded by the European Research Council (StopAutoimmunity, no. 802544).

Conflict of interest

The authors declare no competing interests.

Please refer to the accompanying ICMJE disclosure forms for further details.

Authors' contributions

Conceptualization: Lukas Bajer, Monika Cahova, Johannes R Hov; Methodology: Monika Cahova, Kristian Holm, Petra Polakovicova; Formal analysis, Petra Polakovicova, Filip Tichanek, Kristian Holm; Funding acquisition, Lukas Bajer, Monika Cahova; Investigation, Marie Heczkova, Alena Bohdanecka, Malin H Meyer-Myklestad, Asle W. Medhus, Dag Henrik Reikvam, Mikal J. Hole, Kristin K. Jørgensen; Visualization, Petra Polakovicova; Writing – original draft, Monika Cahova, Petra Polakovicova; Writing – review & editing, Johannes R. Hov, Lukas Bajer.

Data availability

Sequencing data of Czech cohort are available at the Sequence Read Archive database, accession number PRJNA1250244. All scripts are available at https://github.com/xpolak37/PSC_study.

Ethics statement

The Czech study was approved by the Ethics Committee with multi-center competence for IKEM and Thomayer Hospital (reg. no. 13869/20). The Norway study was approved by the Regional Committee for Medical and Health Research Ethics (projects 2015/2140 and 2016/1690). Informed consent was obtained from all subjects involved in the study.

Acknowledgement

The authors would like to thank Mrs. Katerina Dvorakova for her invaluable administrative support and dedication to coordinating this project.

Supplementary data

Supplementary data to this article can be found online at https://doi.org/10.1016/j.jhepr.2025.101716.

References

[1] Karlsen TH, Folseraas T, Thorburn D, et al. Primary sclerosing cholangitis - a comprehensive review. J Hepatol 2017;67(6):1298–1323.
[2] Hov JR, Karlsen TH. The microbiome in primary sclerosing cholangitis: current evidence and potential concepts. Semin Liver Dis 2017;37(4):314–331.
[3] Hov JR, Karlsen TH. The microbiota and the gut-liver axis in primary sclerosing cholangitis. Nat Rev Gastroenterol Hepatol 2023;20(3):135–154.
[4] Demir M, Sigal M. Mucosa-associated microbiota alterations in primary sclerosing cholangitis (PSC) before and after liver transplantation-who is calling the shots? Hepatobiliary Surg Nutr 2023;12(5):795–797.

[5] Schneider KM, Kummen M, Trivedi PJ, et al. Role of microbiome in autoimmune liver diseases. Hepatology 2024;80(4):965–987.
[6] Cancado GGL, Deeb M, Gulamhusein AF. Liver transplantation for cholestatic liver diseases: timing and disease recurrence. Hepatology 2025. https://doi.org/10.1097/HEP.0000000000001268.
[7] Lindström L, Jørgensen KK, Boberg KM, et al. Risk factors and prognosis for recurrent primary sclerosing cholangitis after liver transplantation: a Nordic Multicentre Study. Scand J Gastroenterol 2018;53(3):297–304.
[8] Steenstraten IC, Sebib Korkmaz K, Trivedi PJ. Inet al. Systematic review with meta-analysis: risk factors for recurrent primary sclerosing cholangitis after liver transplantation. Aliment Pharmacol Ther 2019;49(6):636–643.
[9] Hole MJ, Jorgensen KK, Holm K, et al. A shared mucosal gut microbiota signature in primary sclerosing cholangitis before and after liver transplantation. Hepatology 2023;77(3):715–728.
[10] Lennard-Jones J. Classification of inflammatory bowel disease. Scand J Gastroenterol 1989;24(sup170):2–6.
[11] Graziadei IW, Wiesner RH, Batts KP, et al. Recurrence of primary sclerosing cholangitis following liver transplantation. Hepatology 1999;29(4):1050–1056.
[12] Kuhn M. Building predictive models in R using the caret package. J Stat Softw 2008;28:1–26.
[13] Xu S, Zhan L, Tang W, et al. MicrobiotaProcess: A comprehensive R package for deep mining microbiome. The Innovation 2023;4(2).
[14] Mallick H, Rahnavard A, McIver LJ, et al. Multivariable association discovery in population-scale meta-omics studies. PLoS Comput Biol 2021;17(11):e1009442.
[15] Friedman JH, Hastie T, Tibshirani R. Regularization paths for generalized linear models via coordinate descent. J Stat Softw 2010;33:1–22.
[16] Wright M, Ziegler A. Ranger: a fast implementation of random forests for high dimensional data in Cþþ and R. arXiv preprint arXiv:1508.04409 2015.
[17] Hechenbichler K, Schliep K. Weighted k-nearest-neighbor techniques and ordinal classification. 2004.
[18] Robin X, Turck N, Hainard A, et al. pROC: an open-source package for R and S+ to analyze and compare ROC curves. BMC bioinformatics 2011;12:1–8.
[19] Gevers D, Kugathasan S, Denson LA, et al. The treatment-naive microbiome in new-onset Crohn's disease. Cell Host Microbe 2014;15(3):382–392.
[20] Quraishi MN, Acharjee A, Beggs AD, et al. A pilot integrative analysis of colonic gene expression, gut microbiota, and immune infiltration in primary sclerosing cholangitis-inflammatory bowel disease: association of disease with bile acid pathways. J Crohns Colitis 2020;14(7):935–947.
[21] Quraishi MN, Sergeant M, Kay G, et al. The gut-adherent microbiota of PSC-IBD is distinct to that of IBD. Gut 2017;66(2):386–388.
[22] Rossen NG, Fuentes S, Boonstra K, et al. The mucosa-associated microbiota of PSC patients is characterized by low diversity and low abundance of uncultured Clostridiales II. J Crohns Colitis 2015;9(4):342–348.
[23] Kevans D, Tyler AD, Holm K, et al. Characterization of intestinal microbiota in ulcerative colitis patients with and without primary sclerosing cholangitis. J Crohns Colitis 2016;10(3):330–337.
[24] Sabino J, Vieira-Silva S, Machiels K, et al. Primary sclerosing cholangitis is characterised by intestinal dysbiosis independent from IBD. Gut 2016;65(10):1681–1689.
[25] Kummen M, Holm K, Anmarkrud JA, et al. The gut microbial profile in patients with primary sclerosing cholangitis is distinct from patients with ulcerative colitis without biliary disease and healthy controls. Gut 2017;66(4):611–619.
[26] Bajer L, Kverka M, Kostovcik M, et al. Distinct gut microbiota profiles in patients with primary sclerosing cholangitis and ulcerative colitis. World J Gastroenterol 2017;23(25):4548–4558.
[27] Xirouchakis E, Pelekanos A, Xirouchakis S, et al. A systematic review of microbiota in cirrhosis: a change towards a more pathogenic predisposition. Int J Mol Sci 2025;26(2).
[28] Zhang YL, Li ZJ, Gou HZ, et al. The gut microbiota-bile acid axis: a potential therapeutic target for liver fibrosis. Front Cell Infect Microbiol 2022;12:945368.
[29] Trebicka J, Macnaughtan J, Schnabl B, et al. The microbiota in cirrhosis and its role in hepatic decompensation. J Hepatol 2021;75(Suppl 1):S67–S81. Suppl 1.
[30] Bajaj JS, Liu EJ, Kheradman R, et al. Fungal dysbiosis in cirrhosis. Gut 2018;67(6):1146–1154.
[31] Sun LY, Yang YS, Qu W, et al. Gut microbiota of liver transplantation recipients. Scientific Rep 2017;7.
[32] Lu HF, Ren ZG, Li A, et al. Fecal microbiome data distinguish liver recipients with normal and abnormal liver function from healthy controls. Front Microbiol 2019;10.
[33] Vieira-Silva S, Sabino J, Valles-Colomer M, et al. Quantitative microbiome profiling disentangles inflammation- and bile duct obstruction-associated microbiota alterations across PSC/IBD diagnoses. Nat Microbiol 2019;4(11):1826–1831.
[34] Wittek A, Steglich B, Casar C, et al. A gradient of intestinal inflammation in primary sclerosing cholangitis. Inflamm Bowel Dis 2024;30(6):900–910.
[35] Ozdirik B, Scherf M, Brumercek A, et al. Biliary microbial patterns in primary sclerosing cholangitis are linked to poorer transplant-free survival. Hepatol Commun 2023;7(6).
[36] Zigmond E, Zecher BF, Bartels AL, et al. Bile duct colonization with Enterococcus sp. associates with disease progression in primary sclerosing cholangitis. Clin Gastroenterol Hepatol 2023;21(5):1223–12232 e3.
[37] Ozdirik B, Berger H, Tonetti FR, et al. Faecal cytolysin is associated with worse survival in patients with primary sclerosing cholangitis. Liver Int 2025;45(4):e16181.
[38] Nakamoto N, Sasaki N, Aoki R, et al. Gut pathobionts underlie intestinal barrier dysfunction and liver T helper 17 cell immune response in primary sclerosing cholangitis. Nat Microbiol 2019;4(3):492–503.
[39] Ichikawa M, Nakamoto N, Kredo-Russo S, et al. Bacteriophage therapy against pathological Klebsiella pneumoniae ameliorates the course of primary sclerosing cholangitis. Nat Commun 2023;14(1):3261.
[40] Powell CE, McCurry MD, Quevedo SF, et al. Cultured bacteria isolated from primary sclerosing cholangitis patient bile induce inflammation and cell death. bioRxiv 2024. https://doi.org/10.1101/2024.10.08.617321.

Keywords: primary sclerosing cholangitis; recurrence; IBD; mucosal microbiome; liver transplantation; predictive signature; machine learning.

Received 22 July 2025; received in revised form 15 December 2025; accepted 17 December 2025; Available online 22 December 2025

Journal of Hepatology, Volume *8*

Supplemental information

Geography-independent mucosal microbiota alterations in primary sclerosing cholangitis persist after liver transplantation

Lukas Bajer, Petra Polakovicova, Marie Heczkova, Kristian Holm, Mikal J. Hole, Mojmir Hlavaty, Alena Bohdanecka, Pavel Drastich, Filip Tichanek, Malin H. Meyer-Myklestad, Asle W. Medhus, Dag Henrik Reikvam, Kristin K. Jørgensen, Jan Brezina, Peter Macinga, Pavel Wohl, Ondrej Fabian, Johannes R. Hov, and Monika Cahova

Geography-independent mucosal microbiota alterations in primary sclerosing cholangitis persist after liver transplantation

Lukas Bajer, Petra Polakovicova, Marie Heczkova, Kristian Holm, Mikal J. Hole, Mojmir Hlavaty, Alena Bohdanecka, Pavel Drastich, Filip Tichanek, Malin H. Meyer-Myklestad, Asle W. Medhus, Dag Henrik Reikvam, Kristin K. Jørgensen, Jan Brezina, Peter Macinga, Pavel Wohl, Ondrej Fabian, Johannes R. Hov, Monika Cahova

Table of contents

Supplementary methods……………………………………………………………..…...2

Supplementary figures………………………………………………………….……7

Supplementary tables…………………………………………………………..…….9

Supplementary references……………………………………………………………10

Supplementary methods

Study Population

This study is based on two cohorts from Czech Republic and Norway, which consist of 115 non-transplanted PSC (pre_LTx), 159 transplanted PSC (post_LTx) patients, and 96 healthy controls (HCs) Czech subjects underwent colonoscopy between 2021 and 2023 at IKEM, while Norwegian subjects underwent colonoscopy between 2005 and 2008 at Rikshospitalet, Oslo University Hospital. Diagnoses of PSC and IBD were made according to clinical guidelines[1, 2](Karlsen, Lennard). The diagnosis of rPSC was made when cholangiography or histology findings consistent with PSC were present in the absence of defined causes of secondary sclerosing cholangitis[3, 4](Lindstrom, Graziadei). Multiple samples were collected from different gut sites: the terminal ileum in both Czech and Norwegian cohorts, the cecum and rectum in the Czech cohort, and the colon ascendens, colon descendens, and colon sigmoideum in the Norwegian cohorts. For the purpose of comparison and merging of these cohorts, the colon segments were unified under one term - colon. Revised Mayo PSC risk score, Amsterdam–Oxford model (AOM) score for PSC, the aspartate aminotransferase (AST) to platelet ratio index (APRI), and the Fibrosis-4 score (FIB-4) were calculated according to earlier works[5-8] (deVries, Sterling, Wai, Kim). Details are given in **Table 1** and **Supplementary Table 1**.

Sample collection, storage, library preparation, and sequencing

Mucosal biopsies were sampled using standard forceps. PSC samples from the Norwegian cohort were snap-frozen in dry tubes and stored without preservatives, while biopsies from HCs were preserved in RNAlater (ThermoFisherScientific, Waltham, MA); Czech samples were preserved by DNA/RNA Shield (Zymoresearch, Irvine, California, USA). All samples were stored at −80°C until analysis. Fecal DNA from samples in the Czech cohort was isolated by QIAmp PowerFecal DNA Kit (Qiagen), while for Norwegian samples, All Prep DNA/RNA mini kit (Qiagen, Hilden, Germany) was used. Isolated DNA was used as a template in PCR reactions targeting the V3–V4 hypervariable regions of the 16S rRNA gene, using primers 319F/806R for Norwegian samples and 341F/806R for Czech samples, respectively, along with either Q5 High-Fidelity 2× Master Mix (BioLabs, New England) or Phusion High-Fidelity PCR Master Mix with HF buffer. A dual-indexing approach was used. PCR products from Czech samples were cleaned using SPRIselect beads (Beckman Coulter Genomics) and pooled equimolarly according to measured concentrations. PCR products from Norwegian samples were cleaned and normalized using the SequalPrep Normalization Plate Kit (Thermo Fisher Scientific). Quality control and quantification of pooled libraries were performed using an Agilent Bioanalyzer (Agilent Technologies, Santa Clara, CA). Sequencing of Czech DNA libraries was conducted at the Biocev Core Facility (OMICS-Genomics Biocev, Vestec, Czech Republic) on the Illumina MiSeq platform (2×250 bp). Sequencing of Norwegian DNA libraries was performed at the Norwegian Sequencing Centre (Oslo, Norway) on the Illumina MiSeq platform (2×300 bp).

Bioinformatics processing

The Illumina paired-end reads were first quality-checked using FastQC v0.11.9 (https://www.bioinformatics.babraham.ac.uk/projects/fastqc/) and MultiQC v1.12[9]. Nextera Transposase Adapters and PhiX sequences were discarded using BBDuk v39.06 (https://doi.org/10.14806/ej.17.1.200), primers were trimmed by cutadapt v4.9. The reads were then merged using BBMerge v39.06 and oriented with VSEARCH v2.28.1[10]. Amplicon sequence variants (ASVs) were obtained using deblur in QIIME2 version 2024.2[11], after initial trimming to 400bp. The amplicon-region-specific sklearn-based Naive Bayes classifier was trained based on the SILVA reference database[12], release 138.1, with representative sequences at 99% identity via RESCRIPt QIIME 2 plugin. These steps were conducted on a per-run basis, followed by merging the run results using QIIME's feature-table merge, merge-seqs, and merge-taxa functions. Finally, mitochondrial and

chloroplast sequences were filtered out based on the obtained taxonomic assignment. Scripts used to process the raw sequencing data are available at https://github.com/xpolak37/PSC_study.

Statistical analysis

All statistical analyses were conducted using R v4.3.1. The Czech and Norwegian cohorts were merged at the ASV level using custom functions and then divided into two segments: terminal ileum and colon. As the first step, post_LTx, pre_LTx, and HCs samples were analyzed. In the second step, the post_LTx group was further divided into rPSC and non-rPSC groups, with healthy controls also being analyzed. An IBD vs. no-IBD comparison was performed within PSC patients (pre-LTx and rPSC individuals combined). In each step, alpha diversity, beta diversity, differential abundance analysis (DAA) were conducted, and a binary classifier was trained to assess the discriminating power between groups. For the purpose of further calculations, ASVs were aggregated to genus level except alpha diversity, which was calculated at ASV level only. All scripts and analysis reports are available at https://github.com/xpolak37/PSC_study.

Filtering

A rarefaction curve was constructed to determine and visualize suitable sequencing depth for rarefaction using phyloseq package v1.46.0[13]. This was set at 10,000 reads; therefore, all samples below this sequencing depth were removed. The nearZeroVar() function from caret package v6.0-94[14] with default parameters was used to filter low-prevalent and low-abundant taxa. This filtering step was applied to beta diversity, DAA, and classification by the machine learning model, but not for alpha diversity calculations.

Alpha Diversity

Alpha diversity (ASV Richness and Shannon indexes) was calculated on rarefied data at the ASV level. Rarefaction and alpha diversity calculation were performed using the MicrobiotaProcess package v1.14.1[15]. To test differences between groups in the terminal ileum, a linear fixed-effects model was applied (stats package v4.3.1) that accounted for the effect of Country and its interaction with the Group. In the colon dataset, a linear mixed-effect model (robustlmm package v3.3.1)[16] was used, with the Patient variable included as a random effect to account for multiple samples from the same patient, due to merging several segments into a single colon segment. The Benjamini-Hochberg (BH) correction was applied to control the false discovery rate (FDR) for multiple comparisons.

Beta Diversity

The Aitchison distance was calculated using the 'robust_aitchison' method with the vegdist() function in the vegan v2.6.4 package. Permutational MANOVA (PERMANOVA) was performed using the adonis2() (vegan v2.6.4) to assess the effects of Group and Cohort. Initially, the main effects of Group and Cohort were tested using the *by = 'margin'* setting. To evaluate the interaction effect, the model was run once more with *by = 'terms'* setting. When the interaction effect was significant, post-hoc analysis was conducted by separately testing the effect of Group within each Cohort and the effect of Cohort within each Group. In the terminal ileum dataset, PERMANOVA was performed with 999 free permutations. In the colon dataset, 999 custom permutations were used, ensuring that each patient remained assigned to the same group across samples. The BH correction was applied to control the FDR for multiple comparisons. Beta diversity of individual groups was visualized using principal coordinate analysis (PCoA) based on robust Aitchison distance.

Differential Abundance Analysis

Two tools were used for DAA to minimize false positives: linDA from the MicrobiomeStat package v1.2[17] and the Maaslin2 package v1.16.0[18]. The intersection of these two tools was used to identify differentially abundant taxa between groups. From this list, taxa with a significant interaction effect

were excluded based on post-hoc analysis of the Czech and Norwegian cohorts individually. Only taxa with significant log fold change that showed the same direction of change in both cohorts were retained. Taxa associated with the PSC effect were identified by intersecting the differentially abundant taxa from the comparisons between pre_LTx and healthy groups and between post_LTx and healthy groups. Statistics for individual taxa are reported as quartiles of relative abundances for each group and cohort in **Supplementary Tables 2 and 3**. **Supplementary Tables 7 and 8** list the results of DAA, specifically the log fold change, p-values, and FDR values calculated by linDA for each tested comparison.

Classification

Binary classification was performed using Elastic Net (ENET) with glmnet v4.1.8[19] and three supplementary models: Random Forest (RF) with ranger v0.17.0[20], Gradient Boosting (GBoost) with gbm v2.2.2, and K-nearest Neighbors (kNN) with kknn v1.3.1[21]. Hyperparameter tuning was conducted for each model using five-fold cross-validation to estimate the optimal parameters. For the ENET model, the cv.glmnet() function was used to determine the optimal alpha and lambda values (lambda.1se was used). In the other models, the optimal parameters were found using grid search from the caret package. For the GBoost model, tuning was performed for the number of trees (ntrees), interaction depth, shrinkage, and the minimum number of observations in a node (n.minobsinnode). For the kNN classifier, the optimal number of neighbors (k) was tuned. Lastly, the optimal number of variables randomly sampled as candidates at each split (mtry), the minimum node size (min.node.size) was tuned in the RF model. The splitting criterion was set to Gini impurity (splitrule). The tuned parameters, obtained by fitting on the entire dataset, are listed bellow. When validating the models though bootstrapping, the parameters were re-tuned in each iteration.

		ENET		kNN	RF		Gboost			
		alpha	lambda	k	mtry	Min node size	n.trees	interaction depth	shrinkage	n.minobsin node
pre_LTx vs HC	terminal ileum	0.20	0.040	12	47	5	500	1	0.1	20
pre_LTx vs post_LTx	terminal ileum	0.00	0.667	17	57	2	100	1	0.1	10
post_LTx vs HC	terminal ileum	0.00	0.071	12	133	2	200	3	0.1	20
rPSC vs non-rPSC	terminal ileum	0.4	0.312	30	137	2	200	3	0.1	20
rPSC vs non-rPSC (subset)	terminal ileum	0.2	0.502	30	19	5	100	1	0.1	20
pre_LTx vs HC	colon	0.00	0.043	26	23	2	500	5	0.1	20
pre_LTx vs post_LTx	colon	0.00	0.052	15	71	2	500	5	0.1	10
post_LTx vs HC	colon	0.60	0.002	30	65	2	500	3	0.1	10
rPSC vs non-rPSC	colon	0	0.036	12	61	2	500	3	0.1	10
rPSC vs non-rPSC (subset)	colon	0.8	0.033	11	13	2	500	3	0.1	20

These models were trained and validated through bootstrapping (N=500) on CLR-transformed data. Model performance metrics were calculated based on an out-of-bag principle. Bootstrap samples were obtained using sample() function with replacement, with set seed to ensure reproducible results. In the colon dataset, the bootstrap dataset was pseudo-randomly generated to ensure that multiple samples from a single patient were kept in either the training or validation dataset. In all cases, the model was trained using all taxa that remained after preprocessing. For the second objective, distinguish between rPSC and non-rPSC samples, an additional model was trained using only the taxa associated with the rPSC effect identified through DAA. This approach was intended to verify the accurate identification of these taxa and to enhance the model's classification performance.

Classifier performance was evaluated using accuracy metric and the Area Under the Curve (AUC) metric from the Receiver Operating Characteristic (ROC) curve calculated using the pROC package v1.18.5[22]. Optimism-corrected AUC was estimated as the mean AUC from the validation performance across the bootstrapped samples. The lower and upper confidence intervals were calculated using the 2.5th and 97.5th percentiles of the validation AUC distribution. To prevent potential training errors and overfitting, a validity check was performed for all models by randomly shuffling the sample labels. The performance of these models should not reach significantly high values. As expected, the AUC did not exceed 0.57.

Microbial Dysbiosis Index

The Microbial Dysbiosis index (MDI) was defined as clr(total abundance of taxa increased in PSC) – clr(total abundance of taxa decreased in PSC), separately for ileum and colon samples at genus level. Differences between groups were tested using a linear fixed-effects model in the terminal ileum and a linear mixed-effects model in the colon. In the mixed-effects model, the 'Patient' variable was included as a random effect to account for multiple samples from the same patients, same approach like in alpha diversity analysis.

Correlation Analysis

Clinical parameters associated with the severity of gut and liver conditions, as well as other relevant factors, were selected for analysis. The Spearman's correlation coefficient was calculated between the MDI and clinical parameters to assess the relationship between the gut and liver disease severity. For the colon samples, the correlation was computed 100 times, with each calculation performed on a randomly selected sample from each patient. The final reported correlation represents the average across all iterations. A correlation was considered significant if at least 90 out of the 100 iterations yielded a p value < 0.05.

Supplementary figures

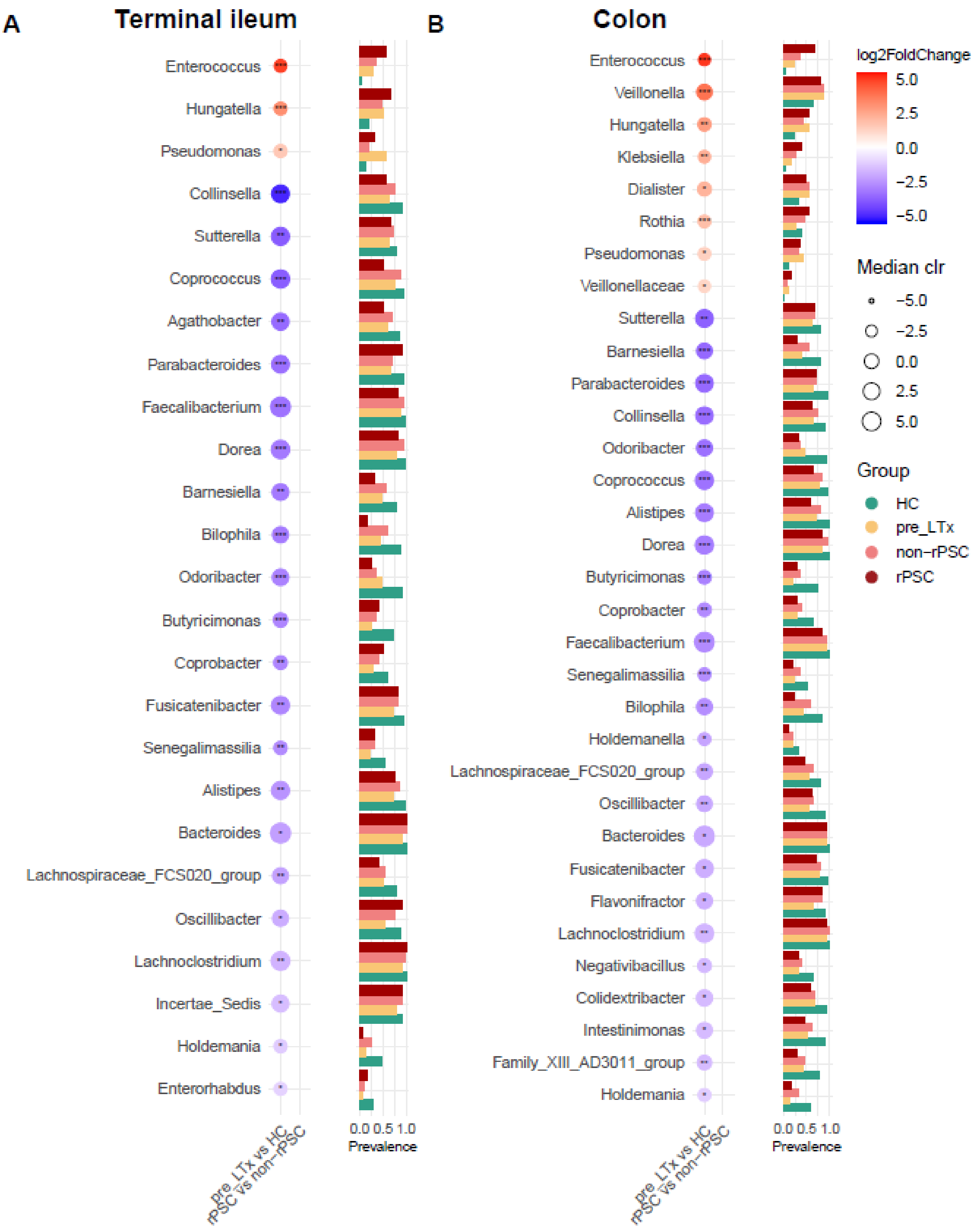

Fig. S1 *Comparison of the relative abundance of bacterial taxa: pre_LTx, post_LTx_rPSC, post_LT_non-rPSC, and HC groups.* (A) Terminal ileum; (B) Colon. The differential abundance analysis was performed independently by two tools (linDA and MaAslin 2), and only the intersection of sets selected by each tool was shown to minimize false positives. The prevalence is calculated as a ratio of samples in which the particular taxon is present to the total number of samples in the group. * q<0.05; * q<0.05; ***q<0.001.

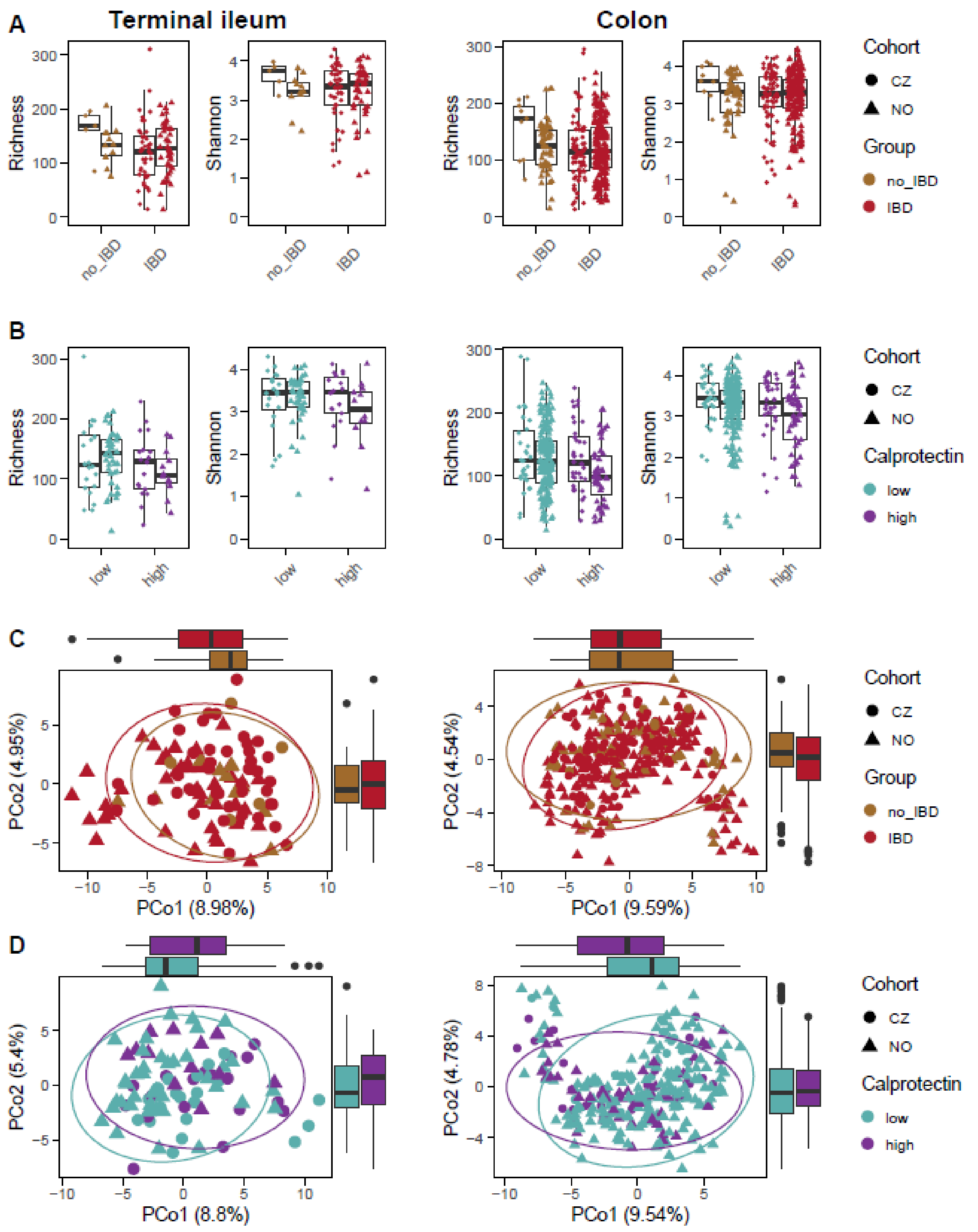

Fig. S2 *Relationship between intestinal inflammation, PSC status and microbiota.* Alpha diversity in the terminal ileum and colon assessed by the Richness and the Shannon indexes based on amplicon sequence variants (ASVs) in (A) IBD and non-IBD groups; (B) low- and high inflammatory groups. Principal coordinate plots showing microbiota composition in (C) IBD and non-IBD groups and (D) low- and high-inflammatory groups. The low and high-inflammatory groups were defined based on fecal calprotectin concentration at a threshold of 250 µg/g.

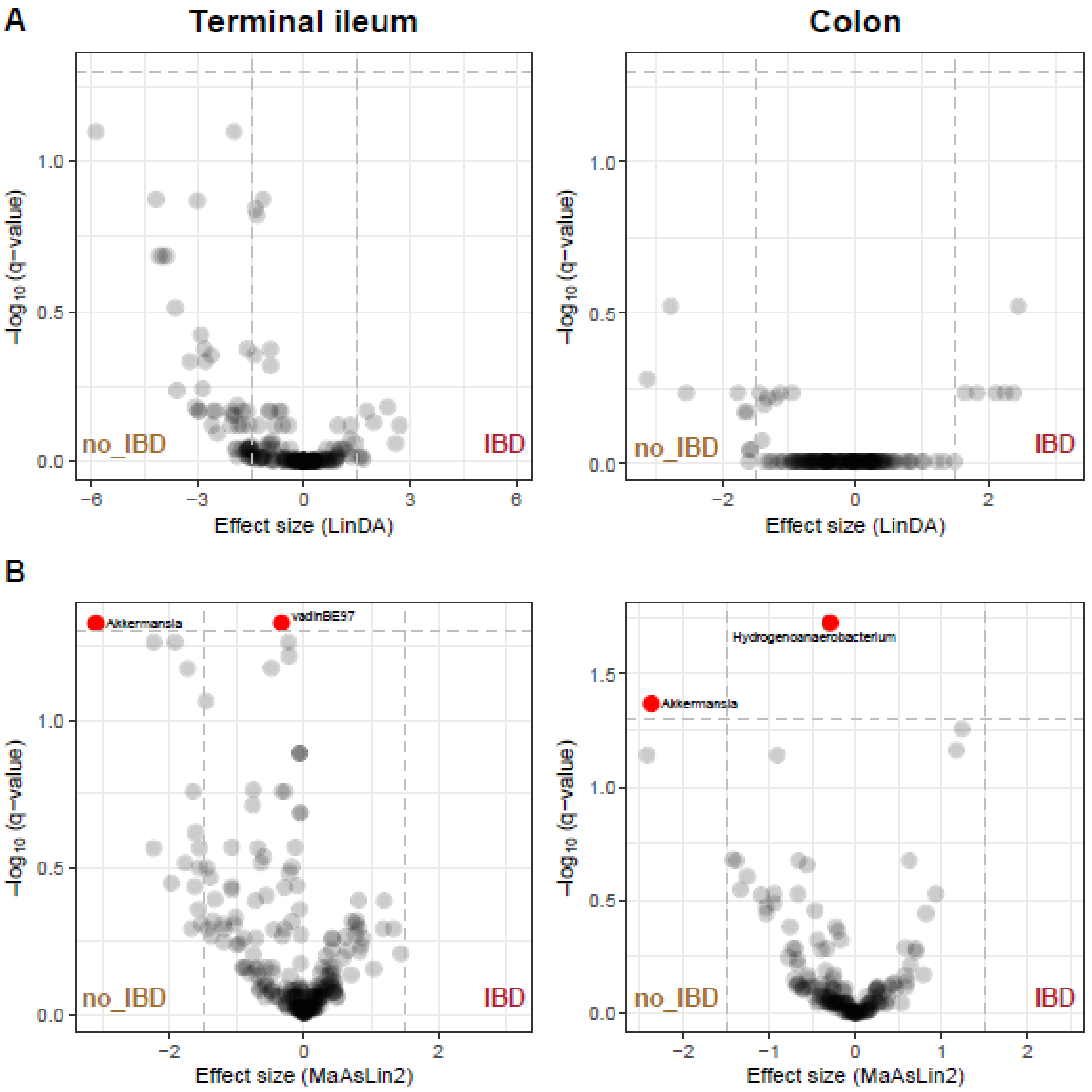

Fig. S3 *Effect of intestinal inflammation on microbiota composition in PSC in terminal ileum and colon.* (A) Volcano plots generated using linDA; (B) Volcano plot generated using MaAsLin2. For this analysis, pre-LTx and post_LTx rPSC patients were pooled and divided according to the history of IBD diagnosis into the PSC_IBD and PSC_noIBD groups. Negative effect size is associated with higher abundance in PSC patients without IBD. The significance threshold was set at $q<0.05$.

Supplementary tables

Available at https://doi.org/10.6084/m9.figshare.29430899

Table S1 Cohort overview
Table S2 Composition of mucosal microbiota in ileum (genus level).
Table S3 Composition of mucosal microbiota in colon (genus level). Table S4 *Effect of the group and cohort on alpha diversity* Table S5 *PERMANOVA results.*
Table S6 *The predictive power of microbiome features to identify the group* Table S7 *PSC-associated mucosal microbiota: terminal ileum* Table S8 *PSC-associated mucosal microbiota: colon*
Table S9 *Effect of the group on alpha and beta diversity: ALD vs PSC post_LTx patients*
Table S10 *Effect of the group and cohort on alpha diversity*
Table S11 *PERMANOVA results*
Table S12 *The predictive power of microbiome features to identify the group*

Supplementary references

1. Karlsen TH, Folseraas T, Thorburn D, et al. Primary sclerosing cholangitis–a comprehensive review. Journal of hepatology 2017;67:1298-1323.
2. Lennard-Jones J. Classification of inflammatory bowel disease. Scandinavian Journal of Gastroenterology 1989;24:2-6.
3. Lindström L, Jørgensen KK, Boberg KM, et al. Risk factors and prognosis for recurrent primary sclerosing cholangitis after liver transplantation: a Nordic Multicentre Study. Scandinavian journal of gastroenterology 2018;53:297-304.
4. Graziadei IW, Wiesner RH, Batts KP, et al. Recurrence of primary sclerosing cholangitis following liver transplantation. Hepatology 1999;29:1050-1056.
5. de Vries EM, Wang J, Williamson KD, et al. A novel prognostic model for transplant-free survival in primary sclerosing cholangitis. Gut 2018;67:1864-1869.
6. Sterling R, Lissen E, Clumeck N, et al. S Sulkowski M, Torriani FJ, Dieterich DT, Thomas DL, Messinger D, Nelson M; APRICOT Clinical Investigators. Development of a simple noninvasive index to predict significant fibrosis in patients with HIV/HCV coinfection. Hepatology 2006;43:1317-25.
7. Wai C-T, Greenson JK, Fontana RJ, et al. A simple noninvasive index can predict both significant fibrosis and cirrhosis in patients with chronic hepatitis C. Hepatology 2003;38:518-526.
8. Kim WR, Therneau TM, Wiesner RH, et al. A revised natural history model for primary sclerosing cholangitis, In Mayo Clinic Proceedings, Elsevier, 2000.
9. Ewels P, Magnusson M, Lundin S, et al. MultiQC: summarize analysis results for multiple tools and samples in a single report. Bioinformatics 2016;32:3047-8.
10. Rognes T, Flouri T, Nichols B, et al. VSEARCH: a versatile open source tool for metagenomics. PeerJ 2016;4:e2584.
11. Bolyen E, Rideout JR, Dillon MR, et al. Reproducible, interactive, scalable and extensible microbiome data science using QIIME 2. Nat Biotechnol 2019;37:852-857.
12. Quast C, Pruesse E, Yilmaz P, et al. The SILVA ribosomal RNA gene database project: improved data processing and web-based tools. Nucleic Acids Res 2013;41:D590-6.
13. McMurdie PJ, Holmes S. phyloseq: an R package for reproducible interactive analysis and graphics of microbiome census data. PLoS One 2013;8:e61217.
14. Kuhn M. Building predictive models in R using the caret package. Journal of statistical software 2008;28:1-26.
15. Xu S, Zhan L, Tang W, et al. MicrobiotaProcess: A comprehensive R package for deep mining microbiome. Innovation (Camb) 2023;4:100388.
16. Koller M. robustlmm: An R Package for Robust Estimation of Linear Mixed-Effects Models. Journal of Statistical Software 2016;75:1-24.
17. Zhou H, He K, Chen J, et al. LinDA: linear models for differential abundance analysis of microbiome compositional data. Genome Biol 2022;23:95.
18. Mallick H, Rahnavard A, McIver LJ, et al. Multivariable association discovery in population-scale meta-omics studies. PLoS Comput Biol 2021;17:e1009442.
19. Friedman JH, Hastie T, Tibshirani R. Regularization paths for generalized linear models via coordinate descent. Journal of statistical software 2010;33:1-22.
20. Wright M, Ziegler A. Ranger: a fast implementation of random forests for high dimensional data in Cþþ and R. arXiv preprint arXiv:1508.04409 2015.
21. Hechenbichler K, Schliep K. Weighted k-nearest-neighbor techniques and ordinal classification. 2004.
22. Robin X, Turck N, Hainard A, et al. pROC: an open-source package for R and S+ to analyze and compare ROC curves. BMC bioinformatics 2011;12:1-8.
